# Network models provide insights into how oriens–lacunosum-moleculare and bistratified cell interactions influence the power of local hippocampal CA1 theta oscillations

**DOI:** 10.3389/fnsys.2015.00110

**Published:** 2015-08-07

**Authors:** Katie A. Ferguson, Carey Y. L. Huh, Bénédicte Amilhon, Frédéric Manseau, Sylvain Williams, Frances K. Skinner

**Affiliations:** ^1^Division of Fundamental Neurobiology, Toronto Western Research Institute, University Health NetworkToronto, ON, Canada; ^2^Department of Physiology, University of TorontoToronto, ON, Canada; ^3^Department of Psychiatry, Douglas Mental Health University Institute, McGill UniversityMontreal, QC, Canada; ^4^Departments of Medicine (Neurology) and Physiology, University of TorontoToronto, ON, Canada

**Keywords:** mathematical model, inhibitory networks, theta rhythm, interneuron, computational model, hippocampus, microcircuit

## Abstract

Hippocampal theta is a 4–12 Hz rhythm associated with episodic memory, and although it has been studied extensively, the cellular mechanisms underlying its generation are unclear. The complex interactions between different interneuron types, such as those between oriens–lacunosum-moleculare (OLM) interneurons and bistratified cells (BiCs), make their contribution to network rhythms difficult to determine experimentally. We created network models that are tied to experimental work at both cellular and network levels to explore how these interneuron interactions affect the power of local oscillations. Our cellular models were constrained with properties from patch clamp recordings in the CA1 region of an intact hippocampus preparation *in vitro*. Our network models are composed of three different types of interneurons: parvalbumin-positive (PV+) basket and axo-axonic cells (BC/AACs), PV+ BiCs, and somatostatin-positive OLM cells. Also included is a spatially extended pyramidal cell model to allow for a simplified local field potential representation, as well as experimentally-constrained, theta frequency synaptic inputs to the interneurons. The network size, connectivity, and synaptic properties were constrained with experimental data. To determine how the interactions between OLM cells and BiCs could affect local theta power, we explored how the number of OLM-BiC connections and connection strength affected local theta power. We found that our models operate in regimes that could be distinguished by whether OLM cells minimally or strongly affected the power of network theta oscillations due to balances that, respectively, allow compensatory effects or not. Inactivation of OLM cells could result in no change or even an increase in theta power. We predict that the dis-inhibitory effect of OLM cells to BiCs to pyramidal cell interactions plays a critical role in the resulting power of network theta oscillations. Overall, our network models reveal a dynamic interplay between different classes of interneurons in influencing local theta power.

## 1. Introduction

The prominent local field potential (LFP) theta rhythm (4–12 Hz) is observed in mammals in a variety of brain structures (e.g., the hippocampus, the prefrontal cortex, the subicular complex, the entorhinal cortex, the amygdala), and is most robustly recorded from the CA1 region of the hippocampus (Buzsáki, 2002). Neuronal firing patterns throughout the brain are correlated with hippocampal theta rhythms (Chrobak and Buzsáki, 1998; Dickson et al., 2003; Pape et al., 2005; Mizuseki et al., 2009; van der Meer and Redish, 2011), and thus globally, theta is often thought to be strongly influenced by hippocampal theta (Battaglia et al., 2011). This hippocampal theta rhythm is thought to play a lead role in spatial navigation and episodic memory (Buzsáki, 2002), and is recorded from the hippocampus during rapid eye movement (REM) sleep and voluntary behaviors such as exploration. As theta power is diminished with memory impairment, and is highest when learning is fastest (see Hasselmo, 2005, for a review on linking behavior to hippocampal theta), it is beneficial to focus on the cellular contributions to it. A number of different inhibitory (GABAergic) interneuron types may be involved in local hippocampal oscillations (Klausberger and Somogyi, 2008), and how critical each cell type is to rhythm generation and to the power of different frequencies is yet to be determined. These interneuron types exhibit a wide diversity of morphologies, synaptic targets, and firing properties (Freund and Buzsáki, 1996; McBain and Fisahn, 2001). Here, we focus on theta power and two major interneuron groups: oriens–lacunosum-moleculare (OLM) interneurons and parvalbumin-positive (PV+) interneurons.

CA1 OLM cells are somatostatin-positive (SOM+) cells that form a major class of hippocampal interneurons. They are thought to play an important role in gating the information flow in the CA1 region (Leão et al., 2012). They have a unique morphology: their cell bodies lie in the stratum oriens layer where they can readily receive local pyramidal cell (PYR) excitation, and their axons project to the stratum lacunosum-moleculare (Freund and Buzsáki, 1996) (the same region in which entorhinal cortical input arrives; Maccaferri and McBain 1995) where they inhibit distal apical dendrites of PYRs. *In vitro* work has demonstrated that OLM cells have intrinsic pacemaking activity, and fire action potentials spontaneously at approximately theta frequencies (Maccaferri and McBain, 1996). The resulting assumption that OLM cells provide the major pacemaking theta signal in hippocampus has been widely incorporated in subsequent work, including many network modeling studies. Through these models, OLM cells have been implicated in playing a leading role in coordinating cell assemblies (Tort et al., 2007), in producing theta oscillations (Gloveli et al., 2005; Rotstein et al., 2005; Orbán et al., 2006) and in cross-frequency coupling (Tort et al., 2007; Wulff et al., 2009). To test the contributions of OLM cells in *in vivo*-like conditions, Kispersky et al. (2012) used a dynamic clamp to inject artificial synaptic conductances following Poisson processes for excitation and inhibition onto OLM cells in rat hippocampal CA1 slices. These artificial synaptic inputs kept the cells in a high-conductance, fluctuation-driven spiking regime near threshold, and would drive the cells to periodically fire, as has been reported *in vivo* (e.g., Destexhe et al., 2003). The traditional view of OLM cells as intrinsic theta pacemakers would imply that, under these conditions, OLM cells should fire at theta frequencies. Surprisingly, the authors observed no theta-frequency firing in the spike trains of OLM cells held in this *in vivo*-like state. However, when the artificial input conductances were modulated at a range of physiologically-relevant input frequencies, it was at theta (8 Hz) that the OLM cells exhibited the greatest amount of modulation. Thus, this work supports the view of OLM cells as effective transmitters of theta inputs, as they preferentially responded with spiking activity phase-locked to theta-modulated inputs. On the other hand, this provided direct evidence against the role of OLM cells in generating pacemaking activity at theta in *in vivo*-like states. In accordance with this finding, Amilhon et al. (2015) showed that in an intact hippocampal preparation that robustly and spontaneously expresses theta rhythms (Goutagny et al., 2009), the optogenetic silencing of SOM+ interneurons had limited effect on intrinsically generated theta oscillations.

Interestingly, in this same intact hippocampal preparation, Amilhon et al. (2015) found that silencing PV+ interneurons strongly affected both the frequency and power of the ongoing oscillations. PV+ interneurons are fast-firing and have been thought to be responsible for the synchronization of large groups of PYRs (Freund and Buzsáki, 1996). They comprise three major types of interneurons: basket cells (BCs), which target the perisomatic region of neighboring PYRs; axo-axonic cells (AACs), which target the axon initial segment; and bistratified cells (BiCs), which target the apical and basal dendrites of PYRs, and co-align with the Schaffer collaterals (SCs) (Buhl et al., 1994; Halasy et al., 1996). These distinct PV+ cell types have been shown to fire preferentially at unique phases of hippocampal theta oscillations in anaesthetized rats and awake mice *in vivo* (Klausberger and Somogyi, 2008; Varga et al., 2014), and thus have the potential to contribute uniquely to hippocampal theta oscillations. We note that while many BiCs are PV+, some have also been found to be SOM+ (Lovett-Barron et al., 2012; Varga et al., 2014).

The poorly understood interactions that interneurons have with other cell types make their contribution to network rhythms difficult to determine experimentally. For example, connections between BiCs and OLM interneurons were only recently identified (Leão et al., 2012). Through these connections, OLM cells may serve to inhibit PYR distal dendrites as well as to inhibit BiCs. In turn, these inhibited BiCs may then lead to a dis-inhibition of the PYR proximal dendrites. How OLM cell and BiC input would be integrated and ultimately affect PYR output in an active network remains unclear.

To parse out how various cellular interactions affect the power of local oscillations, we have developed mathematical models that are tied to experimental work at both the cellular and network levels in an intact hippocampal preparation. Our models uncover the complex interplay between OLM cells and BiCs, identifying regimes in which OLM cells minimally or strongly affect the power of network oscillations. Interactions involving the dis-inhibitory effect of OLM cells onto BiCs to PYRs play a critical role in the power of network theta oscillations. For particular OLM-BiC synaptic balances, the OLM cells' direct influence on PYRs counteracts its indirect dis-inhibitory effect (through the BiCs). In this case, when the OLM cell population is silenced, there is a compensatory effect on network power, and thus minimal change in power. However, in other regimes, the dis-inhibition of PYRs does not balance with OLM cells' direct influence, and thus silencing OLM cells has a stronger effect (an increase in power). The different regimes remain when we consider various strengths and connection probabilities. In this way our models are able to provide a theoretical framework to understand the contribution of different cell types in oscillatory activities and why and how inactivation of particular cell types could result in no change in oscillatory signals.

## 2. Materials and methods

Our network models are derived from an intact *in vitro* hippocampal preparation (Goutagny et al., 2009). The models of the individual cells were developed based on patch clamp recordings from interneurons in this intact preparation, and the network size, connections and synaptic characteristics were estimated directly from the preparation or taken from the literature. As such, our models have a high fidelity relative to the biology.

We note that our focus is on the power, and not on the frequency, of theta oscillations. This allows us to utilize actual excitatory postsynaptic current (EPSC) traces, recorded from putative OLM and PV+ interneurons under voltage clamp in the intact hippocampus *in vitro*, to drive our individual interneurons. In this way, we can simplify our network interactions so that they do not include feedback excitation from PYRs. This simplification is important, as PYR networks on their own may produce complex spiking and bursting behaviors, and thus make the microcircuit too complex (i.e., far too large of a parameter space) to be able to understand how BiC-OLM cell interactions affect network rhythms. Cell firing was then spatially integrated using a passive PYR model to generate an LFP representation. To determine how the interactions between OLM cells and BiCs affect local theta rhythms, we explored how specific features of the network affected model LFP power. In addition, we examined silencing of the different cell populations to understand and predict the contributions of each cell type and the connections between them to network theta oscillations.

### 2.1. Experiment

#### 2.1.1. Animals

Animals of both sexes (P20-28) were used. In order to visualize PV+ and SOM+ interneurons, transgenic mice were used where a fluorescent protein tdTomato was expressed under the control of the PV or SOM promoter (PV-tdTomato and SOM-tdTomato mice). To generate PV-tdTomato and SOM-tdTomato mice, PV-Cre mice (B6;129P2-Pvalb^tm1(cre)Arbr^/J, stock number: 008069, the Jackson Laboratory) and SOM-Cre mice (strain name: STOCK Sst^tm2.1(cre)Zjh^/J, from Dr. Josh Huang, Cold Spring Harbor Laboratory) were mated with a reporter mouse line, Ai9 homozygote mice allowing tdTomato expression in Cre-positive neurons (strain name: B6;129S6-Gt(ROSA)26Sor^tm9(CAG−tdTomato)Hze^/J, stock number: 007905, the Jackson Laboratory) in order to produce PV-tdTomato and SOM-tdTomato offspring. Using immunohistochemistry we confirmed that in PV-tdTomato (SOM-tdTomato) mice, the majority of tdTomato+ neurons in CA1 stratum oriens (str. oriens) express PV (SOM) (PV: 87.6%, SOM: 81.5%, in 4 animals), indicating a high degree of specificity in these mice. All animals were treated according to protocols and guidelines approved by McGill University and the Canadian Council of Animal Care.

#### 2.1.2. Intact hippocampal preparation

The acute preparation containing the intact hippocampus was dissected as described previously (Goutagny et al., 2009). Briefly, after decapitation, the brain was quickly removed from the skull and placed in ice-cold high-sucrose solution, containing (in mM) 252 sucrose, 24 NaHCO_3_, 10 glucose, 3 KCl, 2 MgCl_2_, 1.25 NaH_2_PO_4_ and 1 CaCl_2_ (*pH* 7.3, oxygenated with 95% O_2_∕5% CO_2_). From a hemisected brain, the septum and hippocampus along with the interconnecting fibers were carefully and rapidly dissected out using microspatulas. The preparation was trimmed with fine scissors to remove any remaining cortical tissue and the septum was cut off. The intact hippocampal preparation was left to rest with the CA1 side facing up in an oxygenated room-temperature high-sucrose solution (1 *mM* CaCl_2_) for 30 min-1 h before recording. The intact preparation from only one hemisphere was used for each animal, and the preparation from either the left or the right hemisphere was chosen randomly for each experiment.

#### 2.1.3. Electrophysiological recordings and tdTomato labeling visualization

All electrophysiological recordings were performed at 30 ± 2°C, using artificial cerebral spinal fluid (aCSF) containing (in *mM*) 126 NaCl, 24 NaHCO_3_, 10 glucose, 4.5 KCl, 2 MgSO_4_, 1.25 NaH_2_PO_4_, 0.4 ascorbic acid and 2 CaCl_2_ (*pH* 7.3, oxygenated with 95% O_2_ −5% CO_2_). The intact hippocampal preparation was placed and stabilized in the recording chamber using lead weights. PV+ and SOM+ interneurons located in CA1 str. oriens/alveus within the middle hippocampus were recorded using the visually guided whole-cell patch-clamp technique. Prior to recording, neurons were identified by tdTomato labeling in the soma by illuminating with a 554-nm wavelength light using a fluorescence system (PTI, Monmouth Junction, NJ). The electrophysiology setup was equipped with an upright BX51W1 Olympus microscope, a 20x water-immersion objective, Nomarsky optics, an infrared camera (Cohu, San Diego, CA), a monochrome digital camera for fluorescence imaging (DAGE-MTI, Michigan City, IN) and a temperature controller (model TC-324B, Warner Instruments, Hamden, CT). Patch pipettes (2.5 − 4 *M*Ω) were pulled from borosilicate glass capillaries (Warner Instrument, Hamden, CT) and filled with intrapipette solution containing (in *mM*) 144 K-gluconate, 10 HEPES, 3 MgCl_2_, 2 Na_2_ATP, 0.3 GTP, 0.2 EGTA, adjusted to *pH* 7.2 with KOH. An Axopatch-1C amplifier (Axon Instruments, Foster City, CA), a microelectrode AC amplifier (A-M Systems, Sequim, WA), a Humbug 60 Hz noise eliminator (Quest Scientific, Vancouver, Canada), an audio monitor (A-M Systems) and pClamp9 software (Molecular Devices, Sunnyvale, CA) were used for recording. All drugs were obtained from Sigma-Aldrich (St. Louis, MO) unless otherwise noted. Recordings were kept for analysis only if spikes overshot 0 mV (before junction potential correction) and access resistance was < 30 *M*Ω.

For examining intrinsic properties, the oxygenated aCSF was perfused at a relatively fast rate of 20−25 *ml*∕*min* to ensure the health of the preparation and synaptic blockers were used to inhibit synaptic events [5μ*M* 6,7-Dinitroquinoxaline-2,3-dione disodium salt (DNQX), 5μ*M* bicuculline and 25μ*M* DL-2-Amino-5-phosphonopentanoic acid sodium salt (DL-AP5; Abcam, Toronto, Canada)]. Intrinsic properties of each cell were measured in current-clamp mode following published protocols (Huh et al., 2010). The cell's resting membrane potential was measured once the whole-cell configuration was achieved. While the membrane potential of the cell was held at −60 mV in current clamp, a series of small-amplitude 1 s hyperpolarizing steps (10 *pA* increments) were used to determine the membrane resistance and membrane time constant. A series of 1 s depolarizing current steps (10 and 50 *pA* increments) from the holding potential of −60 mV were applied for frequency-current analysis.

For simultaneous LFP and whole-cell recording, the oxygenated aCSF was perfused without synaptic blockers at a rate of 20–25 ml/min, which has been tested to be ideal for generation of network theta oscillations in the intact hippocampal preparation (Goutagny et al., 2009). For LFP recordings, a borosilicate-glass field electrode (≤1 *M*Ω) was placed in CA1 stratum radiatum (str. rad.) of middle hippocampus. Once a stable network theta rhythm was detected, whole-cell recordings were performed on PV+ and SOM+ interneurons located in CA1 str. oriens. For whole-cell recordings, pipette resistance of 2.5 − 4 *M*Ω was used. The junction potential was estimated at −15.2 mV, and membrane potentials were corrected for this. Once a stable whole-cell mode was achieved, access resistance and the neuron's resting membrane potential were noted. Then, the cell was recorded at this resting potential together with the LFP signal (containing network theta oscillations) for 60 s, to observe the neurons spontaneous firing behavior. Next, the neuron's basic properties were quickly checked for, including firing rate, action potential properties, and sag amplitude. Access resistance and resting membrane potential were checked every 5−10 *min* throughout the recording of the cell. Recordings were kept for analysis only if the LFP signal contained oscillations with frequencies exceeding 2.5 Hz.

The reversal potential for inhibitory postsynaptic currents (IPSCs) was determined using electrical stimulation. For these experiments, aCSF perfusion rate of 7 − 8 *ml*∕*min* was used. A monopolar tungsten microelectrode (WPI, Sarasota, FL) was placed on the surface of CA1 (str. oriens/alveus) in the middle hippocampus. The stimulation parameters were controlled using an isolated pulse stimulator (model A360, WPI). One pulse (25 − 300 μ*A* intensity, 0.1 ms duration) was administered every 10 s. CA1 PV+ and SOM+ interneurons located in the middle hippocampus and close to the stimulating electrode were recorded in whole-cell mode. Neurons were held at various potentials in voltage clamp (−100 to +30 mV) during electrical stimulation to record evoked synaptic currents. To isolate *GABA*_*A*_-receptor mediated IPSCs, 10μ*M* DNQX, 25 μ*M* DL-AP5 and 2 μ*M* CGP 52432 were used to block glutamatergic and *GABA*_*B*_-receptor mediated responses. We determined that the IPSCs reversed around −85 mV (junction potential corrected).

### 2.2. SOM+ cellular model development

#### 2.2.1. Intrinsic properties of SOM+ cells

The intrinsic properties of the nine SOM+ interneurons were determined from whole-cell patch-clamp recordings during the application of synaptic blockers (passive properties for eight cells). The cell's membrane potential was held in current clamp at −60 mV, and a series of small-amplitude 1 s hyperpolarizing steps (10 *pA* increments) were used to determine the input resistance, *R*_*in*_ (*M*Ω), and membrane time constant, τ_*m*_ (*ms*). *R*_*in*_ was calculated by computing the slope of the voltage change over the amplitude of the current injected. τ_*m*_ was derived by fitting the voltage change during a small hyperpolarizing current step with a single exponential function and calculating the mean fit over a few small current steps, such that τ_*m*_ effectively represented the amount of time required for the membrane potential to reach ~63% of the total change. The capacitance was determined by τ_*m*_∕*R*_*in*_. The action potential threshold was set to be the first voltage point such that the slope of the membrane potential exceeded 20 mV/ms (Bekkers and Delaney, 2001), and the spike width was determined at the threshold value. The spike height and the minimum membrane potential reached following the spike were also determined.

The frequency-current (f-I) profiles of the cells are important to characterize, as we aim for our single cell model to respond to a variety of synaptic input strengths with frequencies similar to that observed experimentally. These f-I curves were determined by applying depolarizing current steps of one second duration to cells held in current clamp. Amplitudes were increased incrementally with step sizes of either 50 pA (for five of nine SOM+ cells), or 10 pA (for four of nine SOM+ cells). The frequency (Hz) was determined as the inverse of the average inter-spike interval (ISI) (ms) over the course of the one second step. SOM+ interneurons exhibit adaptation, and thus their firing frequencies differ at the beginning of the 1 s current step than at the end. Thus, for these cells, we determined the initial firing frequency based on the inverse of the first ISI, and the final frequency on the inverse of the last ISI in the 1 s depolarizing step.

For each cell, the approximate linear slope of the f-I curve (and the initial and final f-I curves when relevant) above a threshold frequency was determined using a least squares method, where the threshold frequency was 40 Hz. This value was chosen since above 40 Hz, the slope of the respective cell type was well-approximated by linearization. In addition, the minimum amount of current required to initiate a spike, the rheobase current (*I*_*rheo*_, in *pA*), was determined.

To find the amount of spike frequency adaptation (SFA), we plotted the ISI with respect to the latency of each interval from the start of the current step. The slope of the line fit to these points was used to quantify the amount of SFA (Hemond et al., 2008).

#### 2.2.2. Single cell model structure for SOM+ cells

Similar to our previously developed CA1 fast-firing PV+ interneuron model (Ferguson et al., 2013—see Supplementary Material and Table S1 for details), we develop a simple model of CA1 SOM+ interneurons using Izhikevich's (2003) two dimensional system of ordinary differential equations. Here, we briefly describe the construction of this model, following the methods developed in Ferguson et al. (2014). The Izhikevich model is used since it is relatively simple, but still allows us to choose parameters that are related to biophysical quantities (Izhikevich, 2003).

The Izhikevich (2003) model structure has a fast variable representing the membrane potential, *V* (*mV*), and a variable for the slow recovery current, *u* (*pA*). We used a slight modification of the Izhikevich model in order to reproduce the spike widths. The model is given by:

(1) $$\begin{array}{l} C_{m}\dot{V}=k(V-v_{r})(V-v_{t})-u-I_{j,k}+I_{shift}+{\text{EPSC}}_{\text{k}}\\ \;\dot{u}=a[b(V-v_{r})-u] \end{array}$$

$$\begin{array}{l} \text{if}\;V\geq v_{peak},\;\text{then}\;V\leftarrow c,\;u\leftarrow u+d\\ \text{where}\;k=k_{low}\;\text{if}\;V\leq v_{t},\text{​}k=k_{high}\;\text{if}\;V>v_{t} \end{array}$$

The parameters are as follows:

**Table:** 

*C*_*m*_ (*pF*)	is the membrane capacitance.
*v*_*r*_ (*mV*)	is the resting membrane potential.
*v*_*t*_ (*mV*)	is the instantaneous threshold potential.
*v*_*peak*_ (*mV*)	is the spike cut-off value.
*I*_*j, k*_ (*pA*)	represents the synaptic input from the presynaptic cell population *j* to the postsynaptic cell population *k*.
EPSC_k_ (*pA*)	is the EPSC input to the population *k*, and is derived from the experimental EPSC recordings from the respective cell type in voltage clamp.
*I*_*shift*_ (*pA*)	is a current that shifts the f-I curve laterally to allow the model to easily capture the rheobase current.
*a* (*ms*^−1^)	is the recovery time constant of the adaptation current.
*b* (*nS*)	describes the sensitivity of the adaptation current to subthreshold fluctuations. Greater values couple *V* and *u* more strongly resulting in possible subthreshold oscillations and low-threshold spiking dynamics. Further, the sign of *b* determines whether the effect of *u* is amplifying (*b* < 0) or resonant (*b* > 0).
*c* (*mV*)	is the voltage reset value.
*d* (*pA*)	is the total amount of outward minus inward currents activated during the spike and affecting the after-spike behavior.
*k* (*nS*∕*mV*)	represents a scaling factor. *k* = *k*_*low*_ except when *V* > *v*_*t*_, where *k* = *k*_*high*_ is used to adjust the spike width after the threshold.

The parameters *v*_*r*_, *v*_*t*_, *v*_*peak*_, and *c* were directly based on the intrinsic spike characteristics derived from recordings. Specifically, using the average results over all the recorded cells of one type, *v*_*r*_, *v*_*t*_, *v*_*peak*_, and *c* were set to represent the mean resting membrane potential, threshold potential, peak height, and voltage reset value respectively. *k*_*high*_ was determined such that the width of the action potential from threshold in the model matched the average spike width at threshold in the biological cells.

The adaptation parameters *a* and *d* were determined such that the model produced the amount of adaptation that was determined from the experimental data. The parameters *b*, *k*_*low*_ and *I*_*shift*_ were then varied systematically to determine values in which the slope of the model f-I curve was within the range of slopes determined from the experimental f-I curves, and the rheobase current of the model was equal to the average experimental rheobase current.

Since our SOM+ interneurons exhibited resonant properties, we considered *b*-values such that *b* > 0. We initially held *b* and *k*_*low*_ constant (at 0.2 nS and 0.05 nS/mV), and varied *a* (*ms*^−1^) between 0 and 1 with an initial step size of 0.01, and *d* (*pA*) between 0 and 20 with a step size of 1. Choosing our *a* and *d* parameters that returned the best fits to our initial and final slopes, we then varied *b* between 0.1 and 10, and *k*_*low*_ between 0 and 20 (both with a step size of 0.1). Noting that we required more adaptation, we then returned to vary *a* between 0 and 0.1 with an initial step size of 0.0001, and *d* again between 0 and 20 with a step size of 1.

#### 2.2.3. Experimentally constrained SOM+ model

We first describe the intrinsic properties of the CA1 SOM+ cells from recordings done in the same preparation in which the network recordings were obtained. Then, we construct our single cell mathematical model with these properties to ensure that both our intrinsic and network properties are constrained from the same experimental setting.

##### The experimentally determined SOM+ intrinsic properties

To create our SOM+ interneuron model, we recorded the activity of nine SOM+ interneurons in the str. oriens of the intact hippocampal preparation *in vitro*, and determined their intrinsic properties in the presence of synaptic blockers. The input resistance was 188.0 ± 28.9 *M*Ω which, in combination with the time constant (16.8 ± 2.3 ms), gave a membrane capacitance of 89.7 ± 11.7 *pF*. We determined the resting membrane potential (−62.2 ± 5.2 mV), threshold membrane potential (−53.3 ± 2.9 mV), maximal spike height (6.4 ± 14.1 mV), spike width at threshold (1.3 ± 0.2 ms), and minimal membrane potential (−69.9 ± 3.5 mV). The rheobase current is defined as the minimal amount of current required to elicit a spike. We used a series of depolarizing 10 *pA* steps, as done in four of the nine cells, to precisely determine the rheobase current (−4.8 ± 24.4 *pA*).

**Figure 1:**
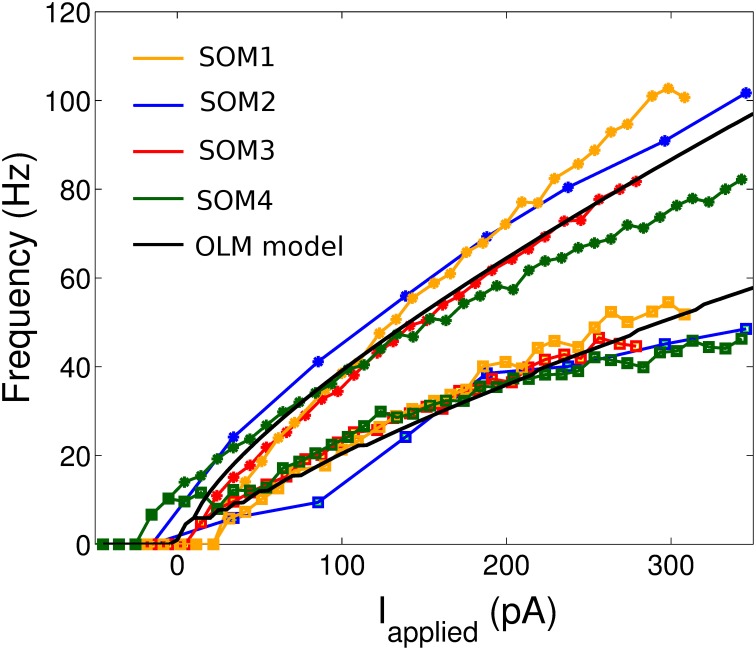
**The initial and final frequency current profiles for four example SOM+ cell recordings (from nine total) in the CA1 region of the intact hippocampal preparation ***in vitro*****. 10 *pA* depolarizing steps were taken for all cells shown except SOM2 (50 *pA* steps). The initial (final) frequencies are shown by asterisks (squares), and the lines interpolate between the data points. The SOM+ cell model, based on the f-I curves of all nine SOM+ recordings, is shown in black.

We then consider the f-I curves of the cell recordings (Figure 1). To demonstrate the amount of adaptation that the cell exhibited, we created two f-I curves for each cell: one based on the first ISI of the cell's spiking during a one second current step (denoted initial curve, data points shown as asterisks in Figure 1), and one based on the last (final curve, data points shown as squares in Figure 1). If the cell only had one spike in the 1 s trace, a frequency of 1 Hz was given.

The values for all these parameters are summarized in Table 1. In this way, our model is constrained with experimentally determined intrinsic properties.

**Table 1:** **Intrinsic properties of CA1 SOM+ (putative OLM) interneurons determined from recordings**.

**Parameter**	**SOM+ cells (n = 9)**
τ_*m*_ (*ms*)	16.80±2.34 (*n* = 8)
*R*_*in*_ (*M*Ω)	187.98±28.87 (*n* = 8)
*C*_*m*_ (*pF*)	89.73±11.67 (*n* = 8)
*v*_*r*_ (*mV*)	−62.2±5.2
*v*_*t*_ (*mV*)	−53.3±2.9
*v*_*peak*_ (*mV*)	6.4±14.1
*c* (*mV*)	−69.9±3.5

##### The experimentally constrained individual SOM+ cell model

We built a simple model of a CA1 SOM+ interneuron using a slight modification of Izhikevich's (2003) two dimensional system of ordinary differential equations, as described in Section 2.2.2.

**Figure 2:**
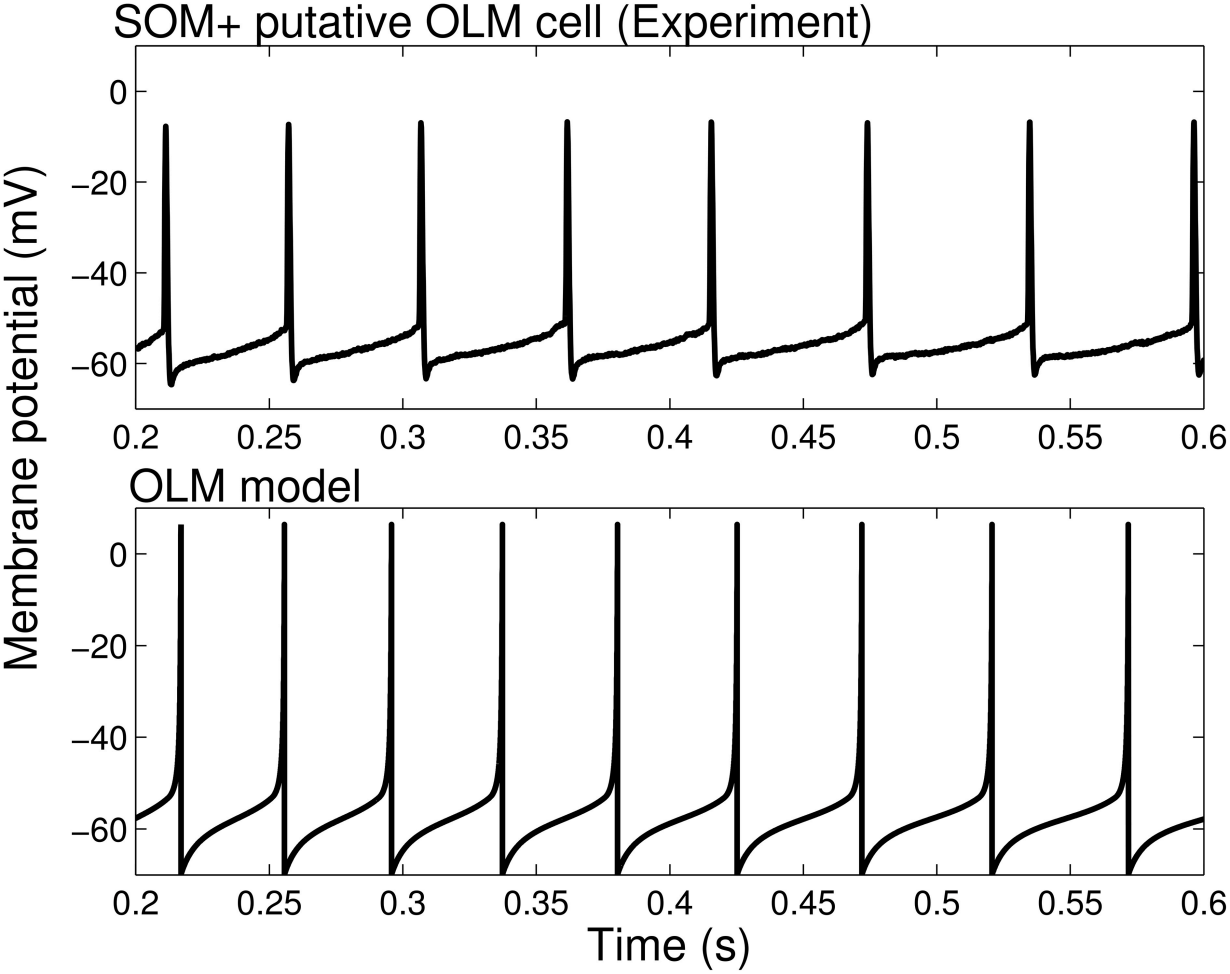
**Firing rates and spike characteristics of SOM+ interneuron model closely matches experiment An example intracellular recording of a SOM+ cell during current clamp with applied current of 61 ***pA*** (top) is compared with the firing of our SOM+ cell model with an applied current of 61 ***pA*** (bottom)**. The spike characteristics and firing rates of the model closely match those of the experiment.

The model parameters were set by our experimentally determined intrinsic properties (as summarized in Table 1), and are given in Table 2. Thus, we set *v*_*r*_ = −62.2 mV, *v*_*t*_ = −53.3 mV, *c* = −69.9 mV, *v*_*peak*_ = 6.4 mV, and *k*_*high*_ = 10 nS/mV in our models. The remaining model parameters were chosen such that the rheobase and initial and final f-I curves of the SOM+ cell model is similar to those of our recordings. Thus, we had to set our membrane capacitance to *C*_*m*_ = 180 *pF*. We determined the rheobase current and the slope of the initial and final f-I curve over 40 Hz (using a least squares approach) for each model in order to settle upon a final model in which our initial and final f-I slopes and rheobase approximated that which we determined biologically. We determined that *a* = 0.0001 ms ^−1^, *b* = 1 nS, *k*_*low*_ = 2, nS/mV and *d* = 2.6 *pA*. This gave us a model f-I initial slope of 0.2422, a final slope of 0.1511, and a rheobase of ~0 *pA* (see Figure 1). As shown in Figure 2, the model firing characteristics are similar to the experimental recordings.

**Table 2:** **SOM+ (putative OLM) model parameters**.

**Parameter**	**SOM+ model**
*C*_*m*_ (*pF*)	180
*v*_*r*_ (*mV*)	−62.2
*v*_*t*_ (*mV*)	−53.3
*v*_*peak*_ (*mV*)	6.4
*a* (*ms*^−1^)	0.0001
*b* (*nS*)	1
*c* (*mV*)	−69.9
*d* (*pA*)	2.6
*k*_*low*_ (*nS*∕*mV*)	2
*k*_*high*_ (*nS*∕*mV*)	10
*I*_*shift*_ (*pA*)	40

### 2.3. Network model development

Here, we describe the rationale for our choice of cell models for the different interneuron types, and the size and connectivity constraints used for our networks. In addition, we describe how we constrained our synaptic parameters using experimentally determined values for the inhibitory reversal potential, the synaptic kinetics, the synaptic conductance strengths, and the overall synaptic drive.

#### 2.3.1. Cell types and rationale for cell models used

The experimental recordings used are from PV+ and SOM+ cells, and our single cell models are of an Izhikevich-type mathematical model structure (see above section). In our network models, we consider four different interneuron types, PV+ BCs, PV+ AACs, PV+ BiCs, and SOM+ OLM cells.

We first note that PV+ BCs and AACs are difficult to distinguish experimentally, and thus we consider them together in our models, and refer to them as “BC/AACs.” We represent them using our previously constructed PV+ cell models (Ferguson et al., 2013) that have the same model structure as in Equation (1). Parameter values are given in Table S1 (Supplementary Material).

We next note that BiCs may not always be PV+ (Lovett-Barron et al., 2012; Varga et al., 2014), but for the work here we assume that they are, as it has been shown in both rats and mice studies (Baude et al., 2007; Katona et al., 2014; Varga et al., 2014). BC/AACs and BiCs share many common properties (Müller and Remy, 2014): they are fast-spiking (Buhl et al., 1996; Varga et al., 2014), their firing is strongly modulated by gamma (30–80 Hz) oscillations *in vivo* (Klausberger and Somogyi, 2008), they respond to gamma rhythmic repeated input in a similar manner (Pouille and Scanziani, 2004), and during sharp waves they receive strong excitatory input (Klausberger et al., 2003, 2004). Although BiCs have also been found to be SOM+ (Lovett-Barron et al., 2012; Katona et al., 2014; Varga et al., 2014), BiCs can be differentiated from SOM+ OLM cells electrophysiologically with their non-accommodating action potentials and ability to exhibit maximum firing rates above 100 Hz (Müller and Remy, 2014). These characteristics were better captured in recordings from our experimental PV+ cells rather than from our experimental SOM+ cells (see Figure 1 and Ferguson et al., 2013). Thus, we represent the BiC populations using our previously developed PV+ fast-spiking model (Ferguson et al., 2013).

Finally, we note that OLM cells are SOM+, although not all SOM+ cells are necessarily OLM cells. However, partial cell reconstructions from SOM+ recordings revealed morphologies that were consistent with OLM cells (Huh et al., in revision), and so we assumed that SOM+ cells were putative OLM cells. The development of our SOM+ (putative OLM) cell model is presented above.

In summary, we consider four different interneuron types, but only two distinct cellular, mathematical models are used. These cellular models will be available on Model DB (http://senselab.med.yale.edu/modeldb/) and Open Source Brain (http://www.opensourcebrain.org/). However, due to cell numbers and connectivity (as described in the next sections), three different interneuron types are distinguished: PV+ BC/AACs, PV+ BiCs, and SOM+ OLM cells.

#### 2.3.2. Network size and cell numbers

Importantly, we need to consider the amount of physical space our network models should represent, and how many individual neuron models are required to represent this space. As described in Ferguson et al. (2013), we previously approximated the minimum circuitry required for theta generation in the CA1 region. It is estimated to comprise a volume of 1 *mm*^3^, and a network of 500 PV+ cells. PV+ BC/AACs target the perisomatic, somatic, and axo-axonic region of neighboring PYRs. However, PV+ BiCs target the proximal apical and basal dendrites of PYRs and have particular connectivity (Leão et al., 2012), and thus may play a distinct role from BCs and AACs. Therefore, in order to connect these populations in distinct ways, we model the PV+ BC/AAC population as separate from BiCs. To determine the cell numbers in each population, we note that approximately 75% of PV+ cells are BC/AACs, whereas 25% are BiCs (Baude et al., 2007). Thus, using a PV+ population of 500 cells, we have a BC/AAC population of 380 cells, and a BiC population of 120 cells. Now, since PV+ interneurons make up 20% of the total population of GABAergic interneurons in the CA1 (Baude et al., 2007), and since SOM+ cells make up 14% of the total GABAergic interneuron population in the hippocampus (Kosaka et al., 1988), our network of SOM+ interneurons is made up of 350 cell models.

#### 2.3.3. Network connections

To determine the connection probability for various cell types, we make estimates based on known connection probabilities, or known divergence of cells and cell numbers, where possible. We describe the probability of a cell in population *j* connecting to a cell in population *k* by *c*_*j, k*_. To determine an approximate range for the number of connections from OLM to PV+ BiCs, we note that 6.3% of connections to PV+ interneurons are inhibitory, and ~2.2% are mutual inhibitory (i.e., PV-PV) connections (Gulyás et al., 1999). So then ~4% of inputs to PV+ cells are from inhibitory sources other than other PV+ cells. Since we estimated that there are 60 connections from other PV+ cells (based on Sik et al., 1995), and twice as many come from other sources, we have 120 connection from non-PV+ interneurons. Thus, we can consider 120 to be our upper bound on the number of connections from OLM-BiC cells. Of course this is considering that all inhibitory connections other than PV-PV connections are from OLM cells, which we know is not true: for example, cholecystokinin-positive (CCK+) cells are an inhibitory class of cells that synapses on PV+ cells (Karson et al., 2009). However, this allows us to have a reasonable range with which we can consider: one BiC receives between 0 and 120 connections from OLM cells. Since we have 350 OLM cells, we examine networks where OLM to BiCs are randomly connected with a probability of *c*_*OLM, BiC*_ = 0−0.33 (with a step size of 0.01 or 0.02). Based on the number of connections found between BiC and OLM cells compared with those of OLM to BiC (Leão et al., 2012), we kept the probability of BiC to OLM connections as 0.64 times the probability of OLM to BiC. As stated previously, our PV+ cells are randomly connected with a probability of 0.12, so that each cell is connected to approximately 60 other PV+ cells (Ferguson et al., 2013).

#### 2.3.4. Inhibitory network model synapses

We used reversal potentials as determined from our experiments (see above) in our model of the synaptic connections (Equation 2). Synaptic input from a cell of population *j* to a cell of population *k* is modeled through a chemical synapse represented by:

(2) $$I_{j,k}=g_{j,k}s_{j,k}(V_{k}-E_{i})$$

where *g*_*j, k*_ (*nS*) is the maximal synaptic conductance of the synapse from a neuron in the presynaptic population *j* to a neuron in the postsynaptic population *k*, *E*_*i*_ is the inhibitory reversal potential. *V*_*k*_ is the membrane potential of the postsynaptic neuron of cell type *k*. The gating variable, *s*_*j, k*_, represents the fraction of open synaptic channels, and is given by first order kinetics (Destexhe et al., 1998; Ermentrout and Terman, 2010):

(3) $${\dot{s}}_{j,k}=\alpha_{j,k}[T_{j,k}](1-s_{j,k})-\beta_{j,k}s_{j,k}$$

The parameters α_*j, k*_ and β_*j, k*_ (in *ms*^−1^) in Equation (3) represent the inverse of the rise and decay time constants respectively, (τ_*rise*(*j, k*)_, τ_*decay*(*j, k*)_ in ms). [*T*_*j, k*_] represents the concentration of neurotransmitter released by a presynaptic spike. Supposing that the time of a presynaptic spike is *t* = *t*_0_, then [*T*_*j, k*_] is represented by a unitary pulse, lasting for 1 ms (until *t*_1_). Then, we can represent

$$s_{j,k}(t-t_{0})=s_{j,k\infty }+(s_{j,k}(t_{0})-s_{j,k\infty })e^{-\frac{t-t_{0}}{\tau_{s(j,k)}}},t_{0}<t<t_{1}$$

where,

(4) $$\begin{array}{ccc} s_{j,k\infty }=\frac{\alpha_{j,k}}{\alpha_{j,k}+\beta_{j,k}} & \text{and} & \tau_{s(j,k)}=\frac{1}{\alpha_{j,k}+\beta_{j,k}} \end{array}$$

After the pulse of neurotransmitter has gone, *s*_*j, k*_(*t*) decays as

(5) $$s_{j,k}(t)=s_{j,k}(t_{1})e^{-\beta_{j,k}(t-t_{1})}$$

When possible, we use experimental estimates of the maximal synaptic conductance values (*g*_*j, k*_) and time constants (τ_*rise*(*j, k*)_, τ_*decay*(*j, k*)_) between cell populations *j* and *k* based on paired recordings in the literature. When exploring the effects of the strength of a connection on network function, we will vary the *g*_*j, k*_ parameter within the experimentally determined range.

To obtain approximate ranges for inhibitory conductance strengths between the interneurons, we turn again to the literature. From Leão et al. (2012), IPSCs from OLM interneurons to BiCs had a peak amplitude of approximately 48 *pA*. Using their same voltage clamp conditions in our cell models (i.e., holding potential at −75 mV, chloride reversal potential at −15 mV, junction potential corrected), an IPSC of a similar amplitude would require a synaptic conductance strength of *g*_*OLM, BiC*_ = 3.2 *nS* (where *g*_*OLM, BiC*_ represents the maximal synaptic conductance from an OLM cell to a BiC. Similarly, for BiC to OLM cell connections, Leão et al. (2012) found IPSCs to be approximately 68 *pA*. Maintaining their voltage clamp conditions in our models resulted in a maximal conductance value of *g*_*BiC, OLM*_ = 3.95 nS. Using these values as initial starting points, we explore the ranges *g*_*OLM, BiC*_ = 0−6 nS and *g*_*BiC, OLM*_ = 0 − 6 nS (with a step size of 0.25 nS).

We chose our recurrent connections between PV+ cells to have a maximal conductance strength of 3 nS, as this was within our experimentally determined values (based on Bartos et al., 2002), and was a value in which our network had the ability to generate coherent oscillations (see Ferguson et al., 2013).

We constrained our synaptic dynamics from the literature. For our PV+ recurrent connections, τ_*rise*(*PV, PV*)_ = 0.27 *ms*, τ_*decay*(*PV, PV*)_ = 1.7 ms based on Bartos et al. (2002). For our OLM-BiC connections, we used τ_*rise*(*OLM, BiC*)_ = 2.6 *ms*, τ_*decay*(*OLM, BiC*)_ = 16.5 ms based on Elfant et al. (2008) recordings of cells in stratum oriens (potential OLM cells) to BCs. As BiC-OLM cells are a relatively newly discovered synapse by Leão et al. (2012), the time dynamics remain unknown. Since Maccaferri et al. (2000) found BiC to PYR connections to have dynamics with a 2 ms rise and 16.1 ms decay (i.e., similar to those found for OLM-BiC; Elfant et al., 2008), we took the connections between BiC and OLM cells to have the same time dynamics.

The network size, connectivity, and synaptic parameters are summarized in Table 3.

**Table 3:** **Network model size, connectivity, and synaptic parameters of the inhibitory OLM-BiC-BC/AAC model**.

	**Number of cells**	**Probability of connection**	**Maximal synaptic conductance (*nS*) OR synaptic weight (to passive PYR (LFP) only)**	**Synaptic time constants**	
				**Rise time (*ms*)**	**Decay time (*ms*)**
BC/AAC	380				
BC/AAC-BC/AAC		0.12	3	0.27	1.7
BC/AAC-BiC		0.12	3	0.27	1.7
BC/AAC-OLM		0	0	0	0
BC/AAC-passive PYR (LFP)		1	0.00067, 0.00038	0.3	3.5
BiC	120				
BiC-BC/AAC		0.12	3	0.27	1.7
BiC-BiC		0.12	3	0.27	1.7
BiC-OLM		0–0.224	0–6	2	16.1
BiC-passive PYR (LFP)		1	0.00067, 0.00044	2	16.1
OLM	350				
OLM-BC/AAC		0	0	0	0
OLM-BiC		0–0.33	0–6	2	16.1
OLM-OLM		0	0	0	0
OLM-passive PYR (LFP)		1	0.00067	3.5	11.8

#### 2.3.5. Experimentally derived excitatory inputs to model cells

Each cell model was driven by EPSCs derived from experimental voltage clamp recordings. Specifically, we chose two recordings, each held at −85 mV (junction potential corrected) to isolate the excitatory synaptic events: one 60 s trace from a PV+ cell, and one from a SOM+ cell. These were used as representative inputs to drive our model cells. Since the EPSCs were recorded while voltage was clamped at −85 mV, we scaled them by a factor of 0.76 to approximate the EPSC amplitudes that would be seen by the cell at rest during theta (~ −68 mV). We subtracted the baseline from these cells so that when no EPSC occurred, the cell did not receive any input overall.

**Figure 3:**
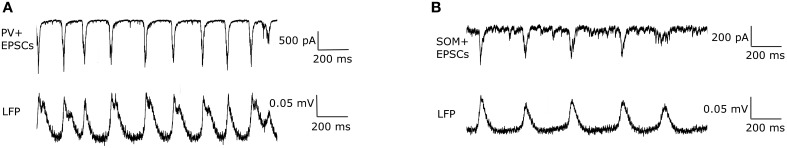
**(A)** An example trace of the EPSCs recorded from a PV+ interneuron (top) during a network theta oscillation (bottom). The EPSCs were recorded under voltage clamp at −85 mV (junction potential corrected), the inhibitory reversal potential, to eliminate IPSCs. Note that the cell receives large excitatory inputs, that are precisely timed to the peak of the LFP. **(B)** Same as in **(A)** but for a SOM+ interneuron. Note the difference in scales: EPSCs received by SOM+ interneurons are smaller than those received by PV+ interneurons.

**Figure 4:**
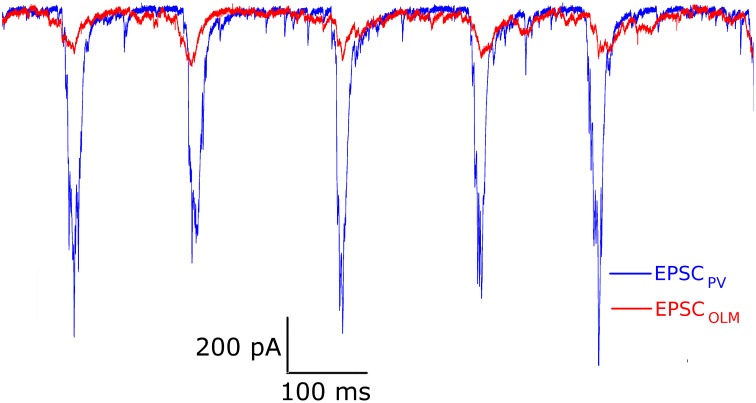
**Example model excitatory postsynaptic current (EPSC) input drives to PV+ and OLM cell models (called EPSC_PV_ and EPSC_OLM_, respectively), based on a modification of experimental EPSCs from voltage clamp recordings**. Amplitudes and phases were varied to produce firing of PV+ and SOM+ cells as seen in experiment.

**Figure 5:**
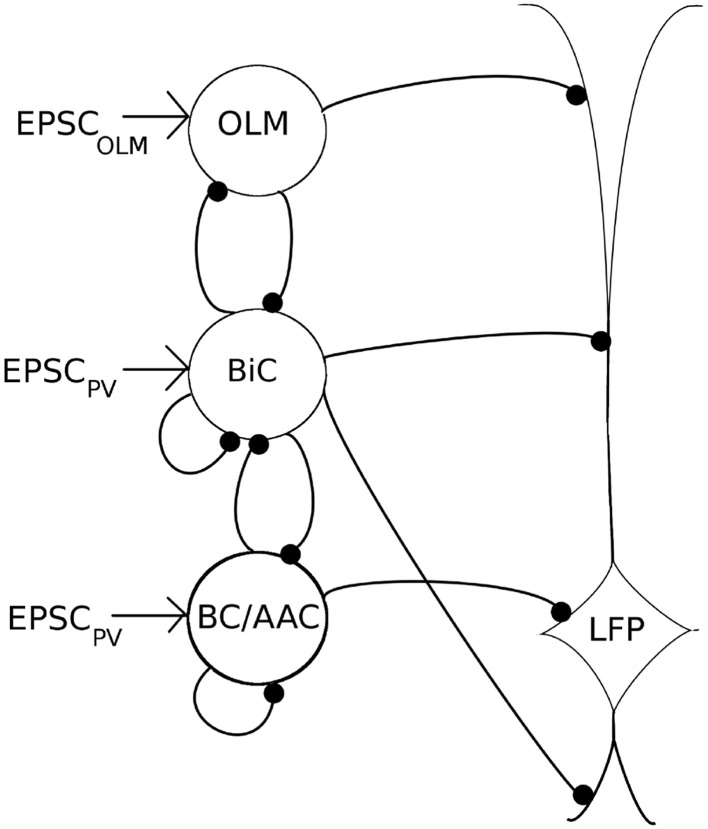
**A schematic of our mathematical network model**. The model contains OLM interneuron models, BiC models, and BC/AAC models. The number of cells (350 OLM, 120 BiC, 380 BC/AAC) and connectivity of each cell type is based on estimates derived from the literature. Filled in black circles represent inhibitory synapses. Each cell receives excitatory input that is taken from our experimental intracellular recordings of the respective cell types (EPSC_PV_ and EPSC_OLM_). Each cell in turn innervates the LFP representation at the appropriate layer. Our LFP representation, which is a somatic recording of a passive PYR model (based on Migliore and Migliore, 2012), integrates these inputs. We use a spectral analysis of the LFP representation to determine the LFP power. As it remains unclear how connectivity between BiCs and OLM cells affects field activity, we have focused on these connections, varying connectivity and connection strength to determine their effects on LFP power.

EPSCs received by PV+ and SOM+ cells are quite precisely timed with respect to the peak of the LFP (Figure 3). However, to target specifically PV+ or SOM+ interneurons, our voltage clamp recordings were done separately on PV-tdTomato and SOM-tdTomato mice, respectively. Thus, to simulate the effect of simultaneously recorded PV+ and SOM+ EPSC recordings, we developed an algorithm to cut and shift the EPSCs. In this way, the two recordings exhibited EPSCs at the same frequency, and the EPSC peaks aligned (Figure 4). We will refer to these frequency-matched currents as EPSC_PV_ and EPSC_OLM_, as shown in Figure 5.

**Figure 6:**
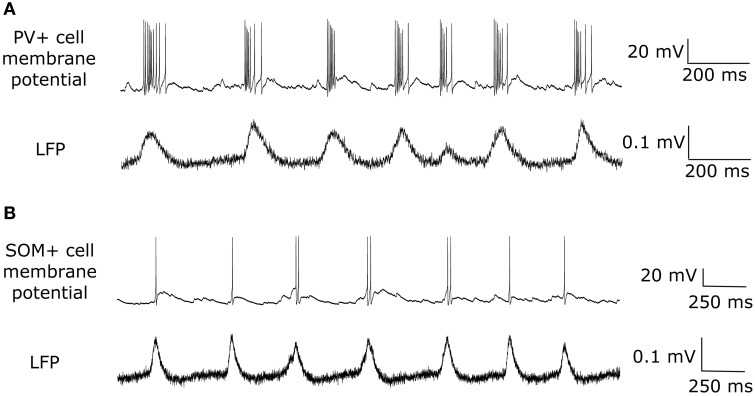
**Experimentally recorded interneurons firing during intrinsically generated theta ***in vitro*****. **(A)** An example one second trace of an experimental recording of a PV+ cell firing (top) during an endogenous theta oscillation (bottom). **(B)** Same as in **(A)** but for a SOM+ putative OLM interneuron.

**Figure 7:**
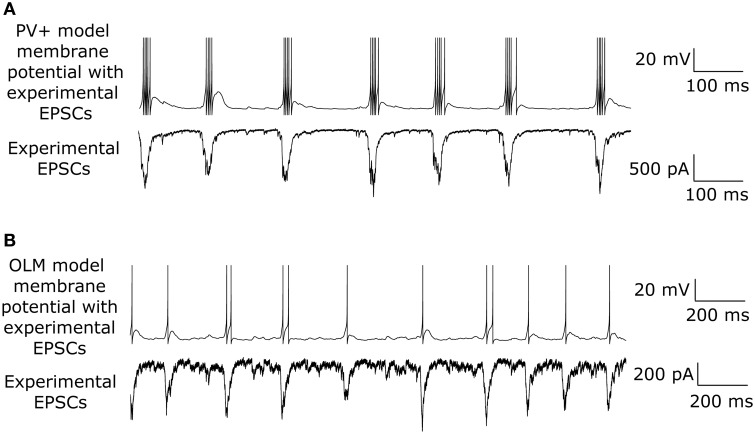
**Model interneurons recapitulate spiking behavior of experimentally recorded interneurons during intrinsically generated theta ***in vitro*****. **(A)** Example one second trace of PV+ cell model firing (top) during the EPSC_PV_ input (bottom). **(B)** Same as in **(A)** but for an OLM interneuron model.

While both PV+ and SOM+ cells showed tendencies to fire slightly before theta peaks, our SOM+ cells (*n* = 9) on average fired 5.32 ms closer to its LFP peak compared to the PV+ cells (n = 8). Thus, we input EPSC_OLM_ 5.32 ms later than EPSC_PV_. To capture the experimental variability in amplitude and timing of EPSCs across cells, we varied the gain (factor by which the EPSC was scaled to alter the amplitude) and timing of the EPSCs across cells with a normal distribution (gain standard deviation: 0.12 for EPSC_OLM_, 0.21 for EPSC_PV_; timing standard deviation: 3.5 ms for EPSC_OLM_, 6.6 ms for EPSC_PV_), in accordance with our experimental recordings. In this way, each cell model received a unique set of excitatory synaptic inputs reflecting the range of amplitudes and timing of those recorded experimentally. In Figure 6 we show example traces of the experimental data, demonstrating PV+ and SOM+ cell firing during an endogenous LFP theta oscillation. We compare this with Figure 7, demonstrating that our cell models fire similarly to the experimental cells when driven with the scaled EPSC_PV_ and EPSC_OLM_ inputs.

#### 2.3.6. Network output—LFP representation

OLM cells, BiCs, and BC/AACs target PYRs at various layers of the hippocampus (stratum lacunosum-moleculare, stratum radiatum/stratum oriens, and stratum pyramidale, respectively), and thus are likely to contribute to the overall network output in unique ways. To provide a representation of the overall network activity, we used a CA1 multi-compartment PYR model to spatially integrate the effects of cell firing across various layers of the CA1 hippocampus. The postsynaptic potentials that occur due to the various cell firings on a passive PYR model give rise to a spatially integrated effect at the soma, a network output, which we consider as our LFP representation. We note that direct excitatory inputs onto the PYR are not included. In addition, we invert the resulting LFP representation so that it can be considered as a very rough approximation of extracellular recordings at the somatic level, or stratum pyramidale. This representation is similar to that used by Tort et al. (2007) in that an excitatory model cell receiving synaptic inputs was analyzed as an LFP output. For the passive PYR model, we used a CA1 PYR model of Migliore and Migliore (2012).

The structure of the Migliore and Migliore (2012) model was based on a morphological 3-D reconstruction of a CA1 PYR. The model was composed of 155 compartments, and was implemented in the NEURON Simulator (Carnevale and Hines, 2006). The passive properties were modeled as *I*_*L*_ = *g*_*L*_(*V* − *E*_*L*_), where *I*_*L*_ is the leak current, *g*_*L*_ is the specific membrane conductance (in *S*∕*cm*^2^), *V* is the membrane potential, and *E*_*L*_ (*mV*) is the reversal potential of the leak channel, which is set to the resting membrane potential of −70 mV. The specific membrane capacitance was given as $C_{m}=1.9\;\mu F∕cm^{2}$. The passive properties were validated against a number of experimental findings, including the somatic and dendritic responses of a CA1 PYR to dendritic current injection under physiological conditions. Since we used the model as a passive integrator of cell firing at various layers of the hippocampus, we eliminated the active conductances from the model. In using this multi-compartment model as a passive integrator of cell firings at various layers of the hippocampus, we distinguished between synapses at the distal layer (stratum lacunosum-moleculare), medial and basal layers (stratum radiatum and oriens), and the perisomatic/somatic layer (stratum pyramidale). Thus, we defined distal synapses as those that were > 475 μ*m* from the soma, apical and basal synapses as those that were 50 − 375 μ*m* from the soma, and perisomatic/somatic synapses as those which were < 30 μ*m* from the soma.

We created three lists of components (where each component points to a specific segment of a section), for the possible distal, proximal apical/basal, and perisomatic/somatic synaptic targets. For each individual, presynaptic inhibitory cell model, we randomly chose a synaptic location on the passive CA1 PYR model from the respective list (distal dendrites for OLM cell models, apical/basal dendrites for BiC models, and perisomatic/somatic locations for BC/AACs). Then the spike times from the individual, inhibitory cell models filled a vector, and an artificial spiking cell was defined to generate spike events at the times stored in that vector at the specific location at which that cell creates a synaptic target. NEURON's (Carnevale and Hines, 2006) *Exp2Syn* function defines the synaptic kinetic scheme of the synapse. This function defines a synapse as a synaptic event with exponential rise and decay, that is triggered by presynaptic spikes, and has a specific weight that determines its synaptic strength. The specific time constants and weights of these connections, as well as the inhibitory reversal potential, are defined next.

#### 2.3.7. Cellular influence on network output

We have considered our LFP representation to be given by the integration of all cell inputs into an individual passive PYR model. We chose our inhibitory synaptic weights onto the passive PYR such that the membrane potential at the PYR soma fluctuates between the resting membrane potential and the inhibitory reversal potential. In general, the further away the synapses are from the PYR soma, the more attenuated they would become at the soma, leading to a smaller influence on the LFP representation. Therefore, we consider two cases: either all cells affect the passive PYR model at their respective synaptic locations with the same weight (i.e., OLM-passive PYR: 0.00067, BiC-passive PYR: 0.00067, BC/AAC-passive PYR: 0.00067), or the weight of the synapses of the various cell types are scaled such that each IPSC recorded at the soma has approximately the same amplitude (~ 26 *pA*) (i.e., OLM-passive PYR: 0.00067, BiC-passive PYR: 0.00044, BC/AAC-passive PYR: 0.00038).

To determine the time constants of our synapses, we used values from Maccaferri et al. (2000) for OLM to PYR and BiC to PYR estimates, and Bartos et al. (2002) for BC/AAC to PYR estimates. However, all of their IPSC recordings were done in the soma of PYRs, whereas our synapses were modeled at the soma and throughout the dendritic tree. To account for this, we scaled our synaptic time constants such that at the model soma, they match those determined in their experimental somatic recordings. Thus, we used τ_*rise*(*OLM, PYR*)_ = 3.5 *ms*, τ_*decay*(*OLM, PYR*)_ = 11.8 ms, τ_*rise*(*BiC, PYR*)_ = 2.0 *ms*, τ_*decay*(*BiC, PYR*)_ = 16.1 ms, and τ_*rise*(*BC*∕*AAC, PYR*)_ = 0.3 *ms*, τ_*decay*(*BC*∕*AAC, PYR*)_ = 3.5 ms.

These values, and synaptic parameters of the inhibitory network, are summarized in Table 3, and a schematic of this network is given in Figure 5.

Thus, we simulated interneuron networks with *g*_*OLM, BiC*_ = 0 − 6 nS and *g*_*BiC, OLM*_ = 0 − 6 nS (with a step size of 0.25 nS), *c*_*OLM, BiC*_ = 0 − 0.33 (with a step size of 0.01 or 0.02, where *c*_*BiC, OLM*_ = 0.64 *c*_*OLM, BiC*_), which produced more than 10, 000 independent and unique networks (each containing 850 interneurons and one model LFP). For each of these scenarios, we explored various possibilities for the way in which the interneurons interact with the LFP representation: one in which all cells affect the passive PYR model at their respective synaptic locations with the same synaptic weight, and one in which all cells affect the passive PYR model soma with the same current amplitude. Therefore, overall we simulated more than 20, 000 interneuron networks. For each network simulation, using the fast Fourier transform, the network frequency was defined as the frequency at which there is a spectral peak in the population activity in the last 4.5 s of our 5 s simulations (disregarding the initial 500 ms of transient activity). The network power was defined as the value of the spectral peak.

### 2.4. Simulations

Our experimental data analysis and generation of the experimentally derived (from voltage clamp recordings) excitatory drives (EPSC_PV_ and EPSC_OLM_) was done using custom codes created in MATLAB (MATLAB, 2014). The inhibitory network model runs were done using the Brian simulator (Goodman and Brette, 2009). As the various populations were connected randomly, we used the same random seed across network simulations. The network model spike times were then input into our LFP representation, which was implemented in the NEURON Simulator (Carnevale and Hines, 2006). For networks in which a particular cell population was “silenced,” we eliminated connections to and from that population, including any connections to the LFP representation. In this way, the silenced population had no effect on network output. The initial conditions of our membrane potentials (*V*) were chosen to be uniform random values from −55 to −65 mV. We used the forward Euler method for integration with a time step of 0.001–0.01 ms, and our runs were done for 5 s simulation time. Our simulations were run on the GPC supercomputer at the SciNet High Performance Computing Consortium (Loken et al., 2010) (http://www.scinethpc.ca/).

## 3. Results

To examine how cellular interactions affect theta frequency power, we used our computational network models that were experimentally constrained at both cellular and network levels. We built on our previous PV+ network models (Ferguson et al., 2013) and focused on the interactions between OLM cells and BiCs. We explored (within physiological ranges) how various maximal synaptic conductance strengths and probability of connections affected model LFP power. We did this using our network models that include 350 OLM interneurons, 120 PV+ BiCs, 380 PV+ BC/AACs, and a representation for our network output (denoted our “LFP representation”). Each inhibitory cell receives excitatory input based on EPSC recordings from our PV+ and SOM+ cells *in vitro*. (See Section 2.3 for more details of the network model construction).

We simulated multiple unique networks with maximal synaptic conductance values of *g*_*OLM, BiC*_ = 0 − 6 nS and *g*_*BiC, OLM*_ = 0 − 6 nS (with a step size of 0.25 nS) for several different connectivities. For each of these networks, our simulations produced the firing activity of each cell in the network, and an LFP representation was generated (see Section 2.3, for details). We did a spectral analysis of our LFP representations and used the peak amplitude as a measure of the power of the theta network activity. We note that the peak frequency will not change, since we are imposing a theta oscillation of a fixed frequency on the interneurons through the experimentally derived EPSC drive (EPSC_PV_ and EPSC_OLM_ for PV+ and OLM cells respectively). We also asked how the strength at which these cell types influence the passive PYR model affected overall power of the LFP representation, and simulated inactivation of the different interneuron populations.

### 3.1. Network theta power can be high or low

**Figure 8:**
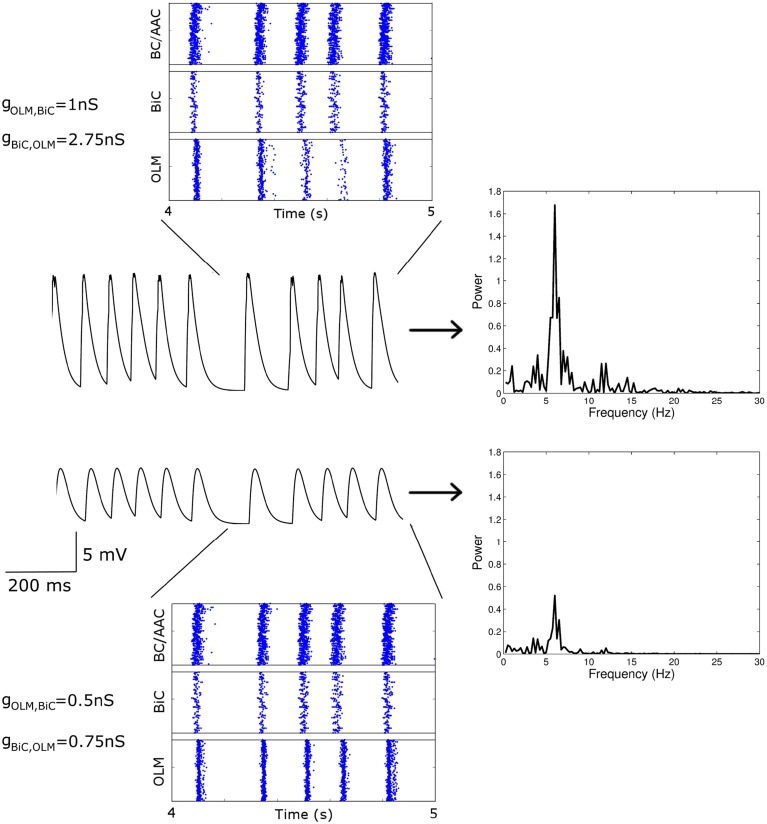
**Two simulated networks with different OLM-BiC synaptic strengths are compared**. The effect of the synaptic strengths between the two cell types (*g*_*OLM, BiC*_ and *g*_*BiC, OLM*_) is difficult to visualize from the raster plot output of the network, whereas the spectral peak power of the LFP output is quite different. Raster plots (left, top and bottom), LFP representation output (left, middle), and resulting LFP power spectrum (right, in *mV*^2^∕*Hz*) are shown for two 850-cell networks with various maximal conductance values for OLM-BiCs (top: 1 nS; bottom: 0.5 nS) and BiC-OLM (top: 2.75 nS; bottom: 0.75 nS). Here the populations are randomly connected with a probability of 0.21 for OLM-BiC connections, and 0.13 for BiC-OLM connections, and each cell type is connected to our passive PYR model with equal connection strength.

From our many simulations, we found that the spectral peak power of the LFP output representation could be high (approx > 1.5 *mV*^2^∕*Hz*) or low (approx < 1 *mV*^2^∕*Hz*). Even when the power was quite different, the effect of the synaptic strengths between the two cell types (*g*_*OLM, BiC*_ and *g*_*BiC, OLM*_) could be difficult to visualize from the raster plot output of the network. This can be seen from Figure 8, where two simulated networks are compared. The first (top) has weaker OLM-BiC connections relative to BiC-OLM ones (*g*_*OLM, BiC*_ = 1 nS; *g*_*BiC, OLM*_ = 2.75 nS), whereas the second (bottom) has overall weaker synapses, and also weaker OLM-BiC connections relative to BiC-OLM ones (*g*_*OLM, BiC*_ = 0.5 nS; *g*_*BiC, OLM*_ = 0.75 nS). The theta power in the top example is high whereas the power is low in the bottom example.

**Figure 9:**
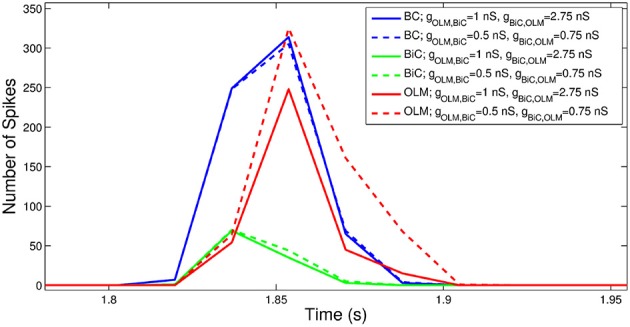
**The number of spikes and the phase of firing differs between networks, depending on the strength of OLM-BiC connections**. Here, the total number of spikes for each cell population during one theta cycle is shown for two different network configurations. The representative networks in Figure 8 are chosen to demonstrate how maximal synaptic conductance values between OLM and BiCs affect the timing and number of spikes in each population (example on top in Figure 8 is represented here with solid lines: *g*_*OLM, BiC*_ = 1 nS, *g*_*BiC, OLM*_ = 2.75 nS; example on bottom in Figure 8 is represented here with dashed lines: *g*_*OLM, BiC*_ = 0.5 nS, *g*_*BiC, OLM*_ = 0.75 nS). Here the populations are randomly connected with a probability of 0.21 for OLM-BiC connections, and 0.13 for BiC-OLM connections, and each cell type is connected to our passive PYR model with equal connection strength. The bin size used is 44 ms, and the plotted point is the endpoint of the bin.

**Figure 10:**
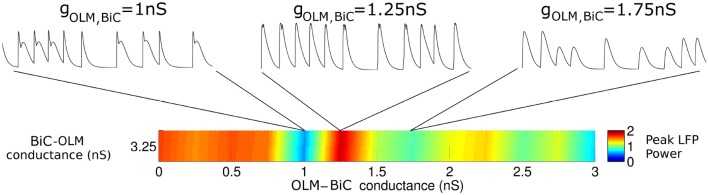
**The interactions between OLM and BiC affect the network power in a complex manner**. Here, a single BiC-OLM conductance (*g*_*BiC, OLM*_ = 3.25 nS) is considered, whereas the OLM-BiC conductances varies, and representative LFP traces are shown. In these simulations, the populations were randomly connected with a probability of 0.19 for OLM-BiC connections, and 0.12 for BiC-OLM connections, and each cell type is connected to our passive PYR model with equal connection strength.

To consider this difference more closely, we considered the distribution of firing of each cell population, and found that the amount of firing and timing of the individual cell populations are factors affecting the power of the network theta oscillations. For the example networks shown in Figure 8, the respective spike distributions of each population are shown in Figure 9 for one of the cycles. It is apparent that the number of spikes and the phase of firing differs between these two networks, particularly for the OLM cell population (this was the case for other cycles for this parameter set). These aspects affect the inputs into the passive PYR model, changing the overall LFP power. Thus, our models indicate that the connection strengths between the two cell populations can strongly influence the amount of firing and their timing (although timing differences are not as large), which ultimately affects network power. Other examples of high and low power also show this difference, but the difference varies for different parameter sets. Precisely how the OLM and BiC populations affect the network power is not straightforward, and depends on the complex balance of interactions between them. An example of how the LFP representation can vary across the OLM-BiC conductance is shown in Figure 10, where the color represents the peak LFP power, and three examples of actual LFP representations are shown. As can be seen, when OLM-BiC conductance strengths increase from 1 nS, there is an increase and then a decrease in power. This suggests that interpreting changes in synaptic strengths (e.g., during plasticity) could be difficult as either increases or decreases in power could be expected. However, it is important to note that the theta power is a metric of the peak LFP spectral power, and it does not capture shape details of the LFP representation. Further, although our LFP representation is able to capture spatio-temporal aspects due to using a multi-compartment PYR model, it is still a simplistic representation relative to the experimental situation.

### 3.2. Cellular interactions and the control of theta power

**Figure 11:**
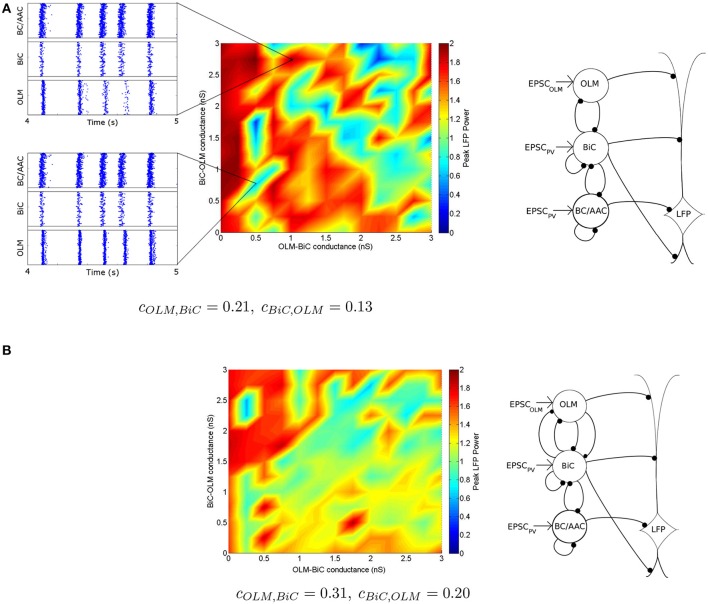
**Distinct regimes in which the network power is affected by connection strengths are apparent**. **(A)** Peak LFP power of our network model (shown in Figure 5) for various connection strengths between OLM and BiCs. The populations are randomly connected with a probability of 0.21 for OLM-BiC connections, and 0.13 for BiC-OLM connections. Each point represents a simulation of our 850 cell network model, where the color of the point represents the peak power of the resulting LFP (see Figure 8 for two example points). Distinct regimes are apparent, where red areas represent higher LFP power, and blue represent lower power. Insets are two example raster plots (top: *g*_*OLM, BiC*_ = 1.0 nS, *g*_*BiC, OLM*_ = 2.75 nS; bottom: *g*_*OLM, BiC*_ = 0.5 nS, *g*_*BiC, OLM*_ = 0.75 nS). Each cell type is connected to our passive PYR model with equal connection strength. On the right, a schematic of the inhibitory network model is shown. **(B)** Distinct regimes in which OLM cells minimally or strongly affect the power of network oscillations remain when the number of connections between OLM and BiCs is varied. OLM cells randomly connect with BiCs with a probability of 0.31, and BiC to OLM cells at 0.20 [as opposed to 0.21 and 0.13 in **(A)**]. Each cell type is connected to our passive PYR model with equal connection strength. On the right, a schematic of the inhibitory network model depicts that the number of connections between BiC and OLM cells are increased.

To compare our networks for *g*_*OLM, BiC*_ = 0 − 6 nS and *g*_*BiC, OLM*_ = 0 − 6 nS simultaneously at a given connectivity, we assign the peak amplitude (theta power) for each network a color (based on the scale shown in Figure 11A), so that we can visualize how changes in synaptic conductance strength result in changes in power. As noted above, this is a particular metric that cannot fully represent the LFP.

With this color visualization, in Figure 11 we can easily see that there are distinct high and low power regimes that come about with different OLM-BiC and BiC-OLM connection strengths—red areas represent higher LFP power, and blue represent lower power. The color plot shown in Figure 11A is for a particular connection probability between OLM and BiC populations (*c*_*OLM, BiC*_ = 0.21; *c*_*BiC, OLM*_ = 0.13).

We also consider the number of (random) connections between OLM and BiCs (*c*_*OLM, BiC*_ = 0.01–0.33, with a step size of 0.02, where *c*_*BiC, OLM*_ = 0.64 *c*_*OLM, BiC*_ based on the number of connections found between these two cell types in Leão et al., 2012—see Section 2.3.3 for details). We find that these distinct regimes remain for various connection probabilities, an example of which is shown in Figure 11B (for the full set of connection strengths, see Figure S1). In all cases, the power still ranged from 0 to 2 *mV*^2^∕*Hz*, indicating that the number of connections between these two cell types is not responsible for producing these distinct regimes, nor does it have a strong effect on theta power: the balance of synaptic strengths appears to be more influential.

**Figure 12:**
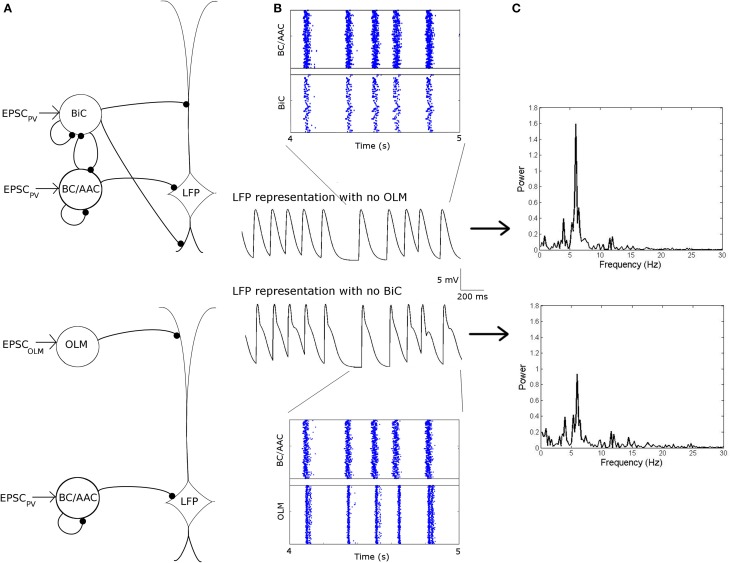
**Networks in which the OLM cells are “silenced” (top) or the BiCs are “silenced” (bottom) are compared**. **(A)** Network schematics for the two networks. **(B)** Raster plots and respective LFP traces of the two networks. Here, each cell type is connected to our passive PYR model with equal connection strength. **(C)** The power of the respective network models. Note the decrease in power when BiCs are silenced as compared to when the OLM are silenced.

To determine a benchmark for what we can consider “high” or “low” theta power in Figure 11, we considered a network in which OLM cells were not present, which can be considered analogous to optogenetically silencing the OLM cells. In this case, our network power was ~1.6 *mV*^2^∕*Hz* (Figure 12, top). This means that the orange/red regions in Figure 11 (and Figure S1) represent networks in which the silencing of OLM cells has little effect on (or a slight decrease in) LFP power, whereas blue regions represent networks in which power is increased (from < 1 to 1.6 *mV*^2^∕*Hz*) when the OLM cells are silenced. Conversely, if BiCs were “silenced” then the network power was ~0.9 (Figure 12, bottom). This means that there could be increases or decreases in network power depending on the precise interactions between the two cell types.

**Figure 13:**
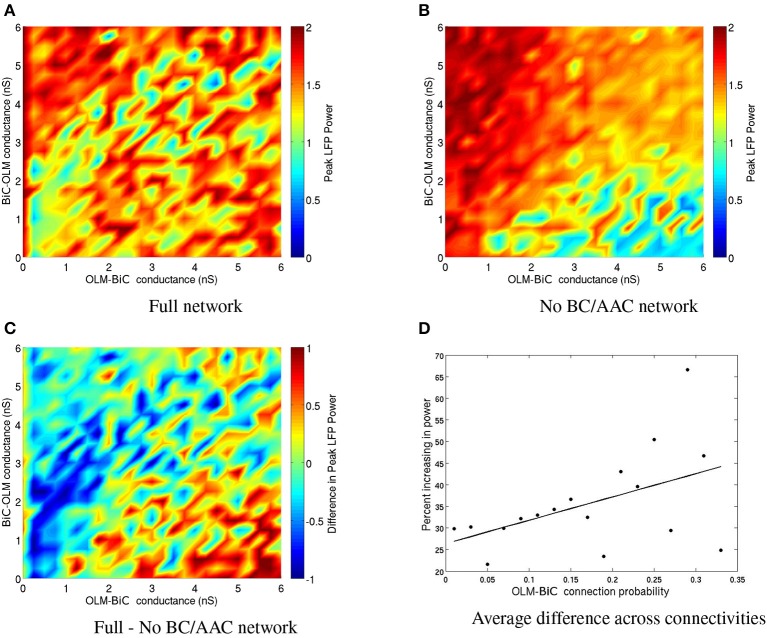
**(A)** Peak LFP power of our network model for various connection strengths between OLM and BiCs. The populations are randomly connected with a probability of 0.25 for OLM-BiC connections, and 0.16 for BiC-OLM connections. Each point represents a simulation of our 850 cell network model, where the color of the point represents the power of the resulting LFP. Each cell type is connected to our passive PYR model with equal connection strength. **(B)** The same simulation as on the left, except that no BC/AACs are present. Note that the overall power is less variable and generally higher, except for the bottom right region. **(C)** The difference between the full network and network with no BC/AACs. Here, warm colors represent an increase in network power when BC/AACs are present. **(D)** The percentage of networks that are increasing upon the addition of BC/AACs across all connectivities.

To consider the effect of silencing BC/AACs, we need to consider the full range of OLM-BiC and BiC-OLM conductance strengths and range of connection probabilities, as these were not tightly constrained. A color plot for a given connection probability in which BC/AACs were silenced is shown in Figure 13B and the full set of connection probabilities are given in Figure S2. Figure 13A shows the full network without any cell types silenced, Figure 13B shows the network when BC/AACs are silenced, and Figure 13C shows the difference between these two. For this connection probability, it can be seen that silencing BC/AACs could result in either increases or decreases in theta power, depending on the particular OLM-BiC and BiC-OLM conductance strengths. This is similar to what we observed with the BiC population. For this particular connectivity example, it can be seen that approximately the lower right diagonal parameter region is where theta power would be decreased by silencing BC/AACs. Considering the full range of connection probabilities as shown in Figure S2, we can say that only at some of the larger connection probabilities (that is, more connections between OLM and BiC populations) does the silencing of BC/AACs lead to a strong effect on theta power (i.e., decreasing it, blue/green regions). This is approximately captured in the plot of Figure 13D where we show that as the number of connections between OLM and BiCs increased, the power did decrease for some larger *g*_*OLM, BiC*_ values. In these cases, our models were more likely to produce a decrease in theta power upon BC/AAC silencing when the connectivity between OLM cells and BiCs was high.

It is interesting to note that when Amilhon et al. (2015) optogenetically silenced PV+ interneurons in the hippocampal preparation, they found that both the frequency and power of the ongoing theta oscillations were diminished. However, when the BC/AAC population was silenced in our network models, the overall power in the network remained high for many but not all BiC-OLM connection strengths (see Figure S2). Given the optogenetic results of Amilhon et al. (2015), we would thus predict that having sufficient connections between these two cell types is critical to the effect of BC/AAC on network theta power, assuming that the silencing of PV+ cells mainly reflects BC/AAC cell types. However, we note that the direct comparison of our models with the optogenetic studies should be viewed with caution as our models lack an active feedback loop from pyramidal cells to interneurons, and are focused on theta power (not frequency).

#### 3.2.1. OLM-BiC interactions play a critical role in the power of network theta oscillations

We note a trend across our simulations: the high and low power regimes are in general distinguished by the OLM-BiC maximal synaptic conductance strength (*g*_*OLM, BiC*_). Lower *g*_*OLM, BiC*_ often leads to higher power, and higher *g*_*OLM, BiC*_ often leads to lower power in general. When OLM to BiC model connections are not too strong, the OLM cells' direct influence on PYRs balances with its indirect dis-inhibitory effect (through the BiCs). In this case, when the OLM cell population is silenced, there can be a compensatory effect, and thus only a minimal change in power occurs. However, when these OLM to BiC connections are stronger, the dis-inhibition of PYRs does not balance with their direct influence, and thus silencing OLM cells has a stronger effect.

As observed above, low and high theta power regimes can be reflected as higher or lower amounts of firing of the OLM cell population respectively (Figure 9). This suggests that for a compensatory effect to be possible, BiC to OLM connections should be strong enough so as to decrease the amount of OLM cell firing. We note that if one were to examine the firing of (i.e., “record” from) the cells on their own, it can be difficult to distinguish differences between the two regimes of high and low theta power (as can be seen by comparing the raster plot insets in Figure 11A). Thus, our models distinguish between regimes in which OLM cells minimally or strongly affect the power of network oscillations, and predict that the dis-inhibitory effect of OLM cells on BiC to PYR interactions plays a critical role in the power of network theta oscillations.

#### 3.2.2. High and low theta power regimes remain when interneuron to pyramidal cell strengths are varied

As described above, the compensatory effect that can occur when OLM cells are silenced so that there is a minimal change in theta power requires OLM cell to BiC connections to not be too strong and BiCs to OLM cell connections to be strong enough. However, such a compensation would also depend on spatio-temporal aspects as captured in the multi-compartment pyramidal cell model with distributed synaptic inputs.

**Figure 14:**
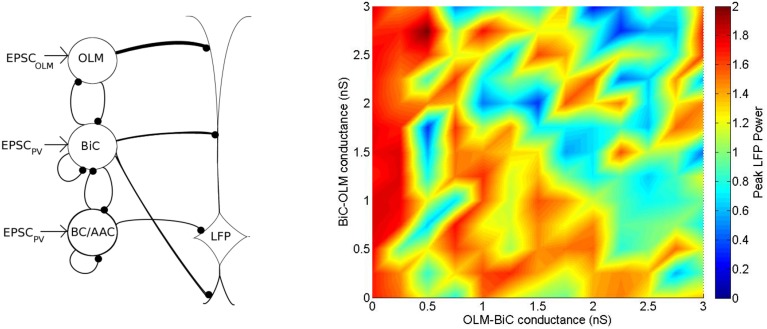
**Left:** A schematic of the inhibitory network model showing that the strength of the connections to the passive PYR model is increased with distance from the soma, in order to create similar input strengths at the level of the soma for all three inputs. **Right:** Distinct regimes in which OLM cells minimally or strongly affect the power of network oscillations remain when the strength at which each cell type influences the passive PYR model is varied. The strength of connections to our passive multi-compartment PYR model is increased with distance, such that each IPSC to the PYR is equal in size (as opposed to Figure 11A in which each connection has equal weights, and thus IPSCs decrease with distance). Here, populations are randomly connected with a probability of 0.21 for OLM-BiC connections, and 0.13 for BiC-OLM connections, as in Figure 11A.

The network model simulations so far have been done with similar synaptic weights between the different interneuron populations and the pyramidal cell (see Table 3). As we do not know precisely how each cell type affects pyramidal cells, and thus our LFP representation, we also consider a varied distribution of strengths in which the cell populations affect the passive PYR model. Specifically, we consider that the strength of connections to our passive PYR is increased with distance from the soma (giving weights of OLM-passive PYR: 0.00067, BiC-passive PYR: 0.00044, BC/AAC-passive PYR: 0.00038), such that each post-synaptic potential onto the passive PYR model is equal in size. This is different from the above simulations, in which each cell population affected the passive PYR model with equal connection strength (giving weights of OLM-passive PYR: 0.00067, BiC-passive PYR: 0.00067, BC/AAC-passive PYR: 0.00067). We find that although overall there is a decrease in LFP power, high and low theta power regimes are still present. Compare Figure 14 with Figure 11A. The full set of connection probabilities are shown in Figure S3. Given the described compensatory effect this makes sense. That is, with the varied synaptic weights in which the further away ones (from OLM cells) are stronger than closer ones, OLM cells' direct influence on PYRs is stronger than for BiCs, and so it would be less likely that a compensatory effect can occur considering the same ranges of OLM-BiC and BiC-OLM cell connection strengths (compare Figure S1 and Figure S3 where less red regions in Figure S3 are apparent).

Thus, the manner in which the interneurons interact with the passive PYR model is of course influential in determining the overall network power. However, the existence of these high and low theta power regimes, which are separated mainly by the strength of the OLM-BiC connections, does not appear to be critically dependent on the strength with which the interneurons influence the passive PYR model.

## 4. Discussion

We constructed our network model with individual cell models of PV+ BC/AAC, PV+ BiC, and SOM+ OLM interneurons, and constrained network size, connectivity, and synaptic properties with experimental data. Experimental EPSCs recorded during endogenous CA1 theta oscillations were used to drive the various cell model populations. To integrate the effects of cell firing, we used an LFP representation, which is the somatic recording of a passive PYR model (Migliore and Migliore, 2012). We focused on investigating how connections between BiCs and OLM cells affect field activity. In particular, we varied the number of connections and connection strengths between these two cell types within physiologically estimated ranges, and the strength at which these cell types influence the passive PYR model. In addition, we simulated silencing of the OLM, BiC, or BC/AAC model populations to understand the effect of cellular interactions on theta power.

Our models distinguish between regimes in which OLM cells minimally or strongly affect the power of network oscillations—high or low theta power in the models respectively. We predict that BiC-OLM connections play a critical role in the power of network theta oscillations. As such, we suggest that more experimental work to characterize these connections could lead to important insight into mechanisms by which theta power is affected physiologically and/or pathologically. These regimes are difficult to distinguish based on the firing pattern of the cell populations alone. In particular regimes, the direct inhibitory influence of OLM cells on PYRs balances with its indirect dis-inhibitory effect (through the BiCs). In this case, when the OLM cell population is silenced, there is a compensatory effect on network power, and thus minimal change in power. However, in other cases, the dis-inhibition of PYRs does not balance with their direct inhibition by OLM cells, and thus silencing OLM cells has a stronger effect. These complex regimes are created in a highly non-linear manner by the balances between OLM and BiCs. This balance affects the precise timing of the various cell populations, in turn affecting how each population influences network activity. Since the density of interneuron types varies across the septo-temporal axis (Jinno and Kosaka, 2006), it is reasonable to assume that the probability of connections, or the connection strengths between OLM and BiCs varies across the axis as well. These differences could result in these cell types influencing network theta oscillations in quite distinct ways. In this way, the entorhinal cortex or the CA3 region may have more or less influence across this axis due to the differential gating of information flow (through the perforant pathway or Schaffer Collaterals, respectively).

We inactivated the different cell populations in our model networks. This is an approximation to optogenetic silencing experiments as we note that our models do not include active feedback. We found that for our results to align with those of Amilhon et al. (2015), where the silencing of PV+ interneurons had a large effect on network theta power, we needed to have a high connection probability between BiC and OLM interneurons. Here we are relating their PV+ silencing to our BC/AAC silencing, which of course does not take into consideration the PV+ BiC population. However, that the BC/AAC population would require particular BiC-OLM interactions to have a large effect on network theta power is not intuitive, and is interesting in itself. We note that eliminating PYR feedback onto the various interneuron populations allowed us to focus on the direct and immediate effects of changes to the microcircuit on the LFP, but of course is a substantial simplification to the network. This can be addressed in the future with a full network model with feedback excitation.

In addition to connection strengths of synapses between OLM and BiCs, we also considered the probability of connections between them. We find that these distinct regimes remain for various connection probabilities. Furthermore, as we do not know precisely how each cell type affects the LFP, we consider various distributions of strengths in which the cell populations affect the passive PYR model, and find that these high and low power regimes in which OLM cells affect LFP power remain. Given our simplistic LFP representation, it is important to incorporate biophysically realistic models of the LFP to represent our network activity (e.g., Einevoll et al., 2013), with the aim of facilitating the comparison between our model and experimental network theta rhythms.

Although we increase our synaptic conductance strengths between OLM and BiCs, our results are based on an estimated balance between these two cell types (based on Leão et al., 2012), which implies that these conductance strengths are kept at a specific ratio. We note that the IPSC recordings from which this proportion is based on was not recorded during theta oscillations, and thus the balance during such an oscillatory network state could be quite different. It would be interesting to explore different balances between these two cell types, and to see how they affect network theta.

During LFP theta oscillations in mice *in vivo*, Varga et al. (2014) demonstrated that CA1 AACs and BCs exhibited theta-modulated firing that preceded BiC theta-modulated firing. However, these groups could not be distinguished from spike timing alone, and required direct anatomical identification (Varga et al., 2014). Our PV+ population did not exhibit any distinct subgroups based on firing properties, nor on the timing of its EPSCs. It remains unclear whether this phase difference exists between PV+ interneuron subclasses in the local CA1 theta rhythm generation recorded in the intact hippocampal preparation *in vitro*, or if this phase difference occurs due to external theta input on the CA1 hippocampus *in vivo* (e.g., the medial septum). Due to these factors, we did not address how this difference in preferred phase of firing between the PV+ populations would affect theta power.

In summary, this paper describes computational network models of CA1 hippocampus that reveals a complex, dynamic interplay between different classes of neurons in determining oscillatory power in the theta range. Our models provide a theoretical framework to understand the contribution of different cell types in oscillatory activities.

## Author contributions

KF: designed and performed research, analyzed data, wrote paper. CH: designed and performed research, analyzed data, wrote paper. BA: designed and performed research, wrote paper. FM: designed and performed research, wrote paper. SW: designed research, wrote paper. FS: designed research, wrote paper.

## Funding

Grant sponsor: Canadian Institutes of Health Research (CIHR); Grant Number: MOP-102573; Natural Sciences and Engineering Research Canada (NSERC); Grant Number: RGPIN-203700.

### Conflict of interest statement

The authors declare that the research was conducted in the absence of any commercial or financial relationships that could be construed as a potential conflict of interest.
