# Supplementary figures and images for: Network models provide insights into how oriens–lacunosum-moleculare and bistratified cell interactions influence the power of local hippocampal CA1 theta oscillations
Source: Front Syst Neurosci. 2015 Aug 7;9:110. doi: 10.3389/fnsys.2015.00110 (PMC4528165; doi:10.3389/fnsys.2015.00110)

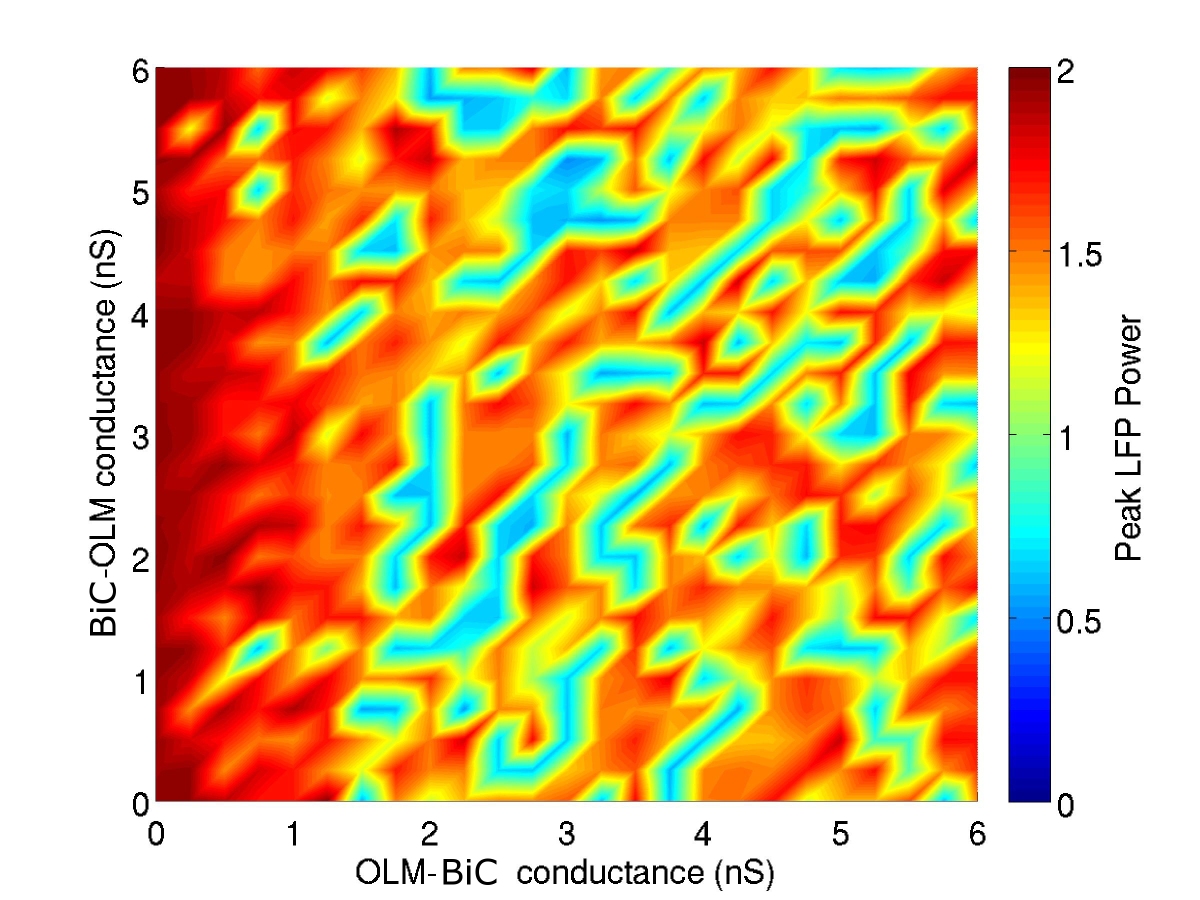

Supplement: Supplementary file 1 [file Presentation1.ZIP › FigS1a.jpg]

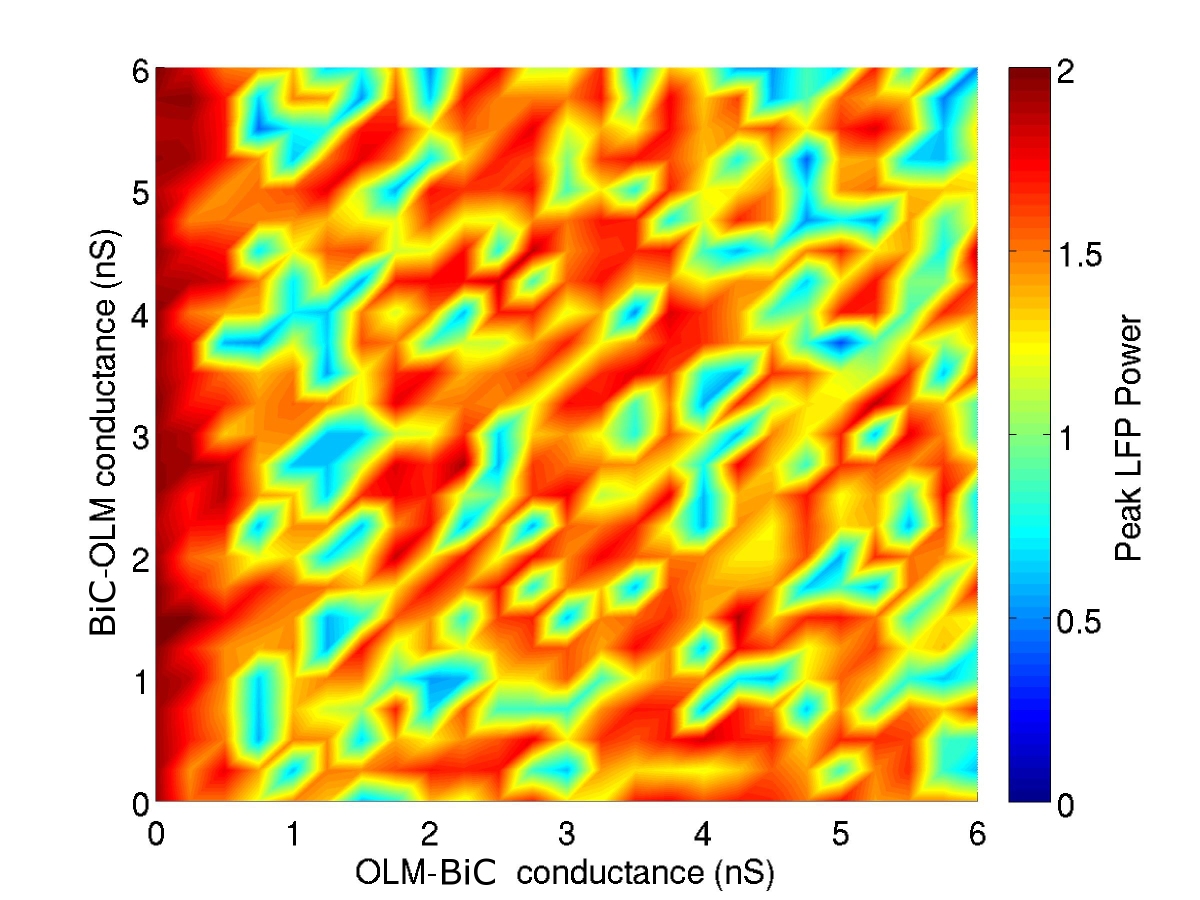

Supplement: Supplementary file 1 [file Presentation1.ZIP › FigS1b.jpg]

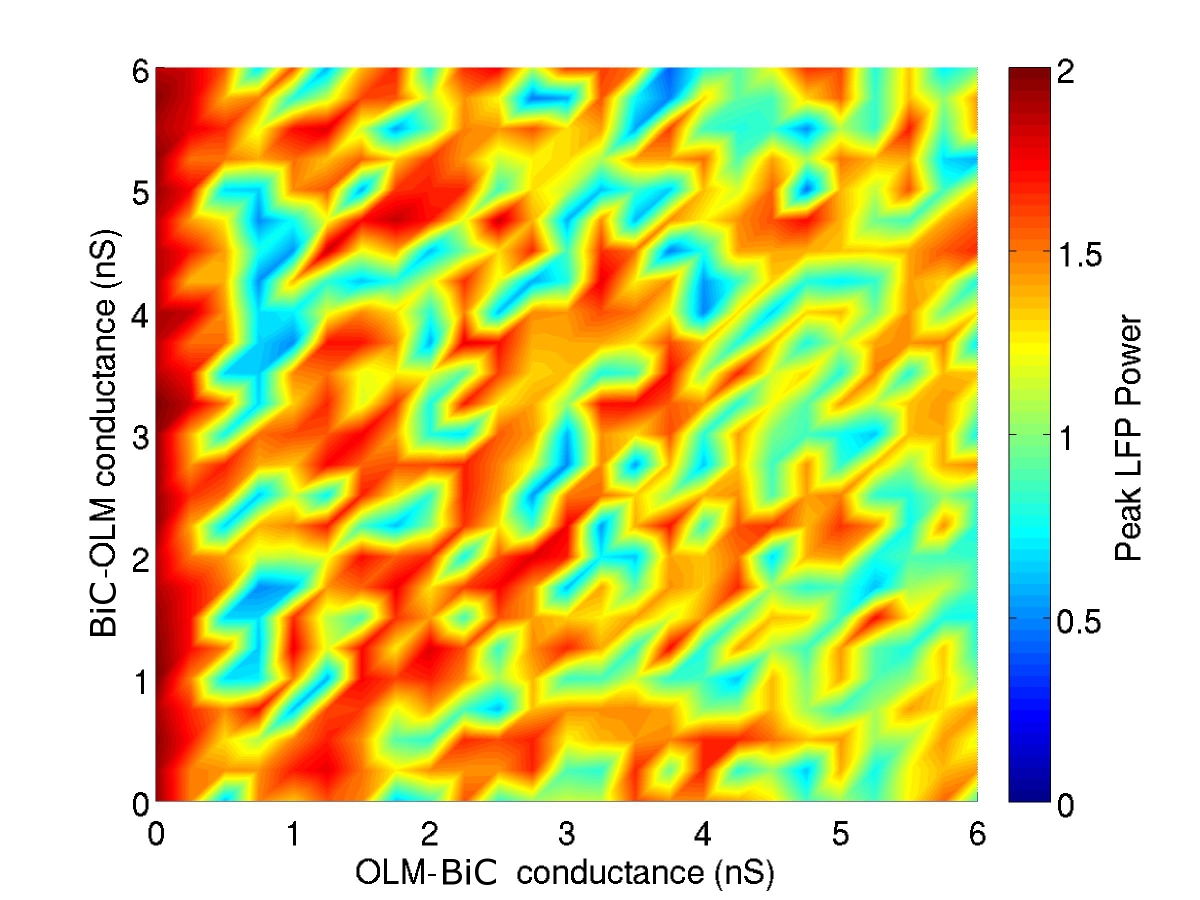

Supplement: Supplementary file 1 [file Presentation1.ZIP › FigS1c.jpg]

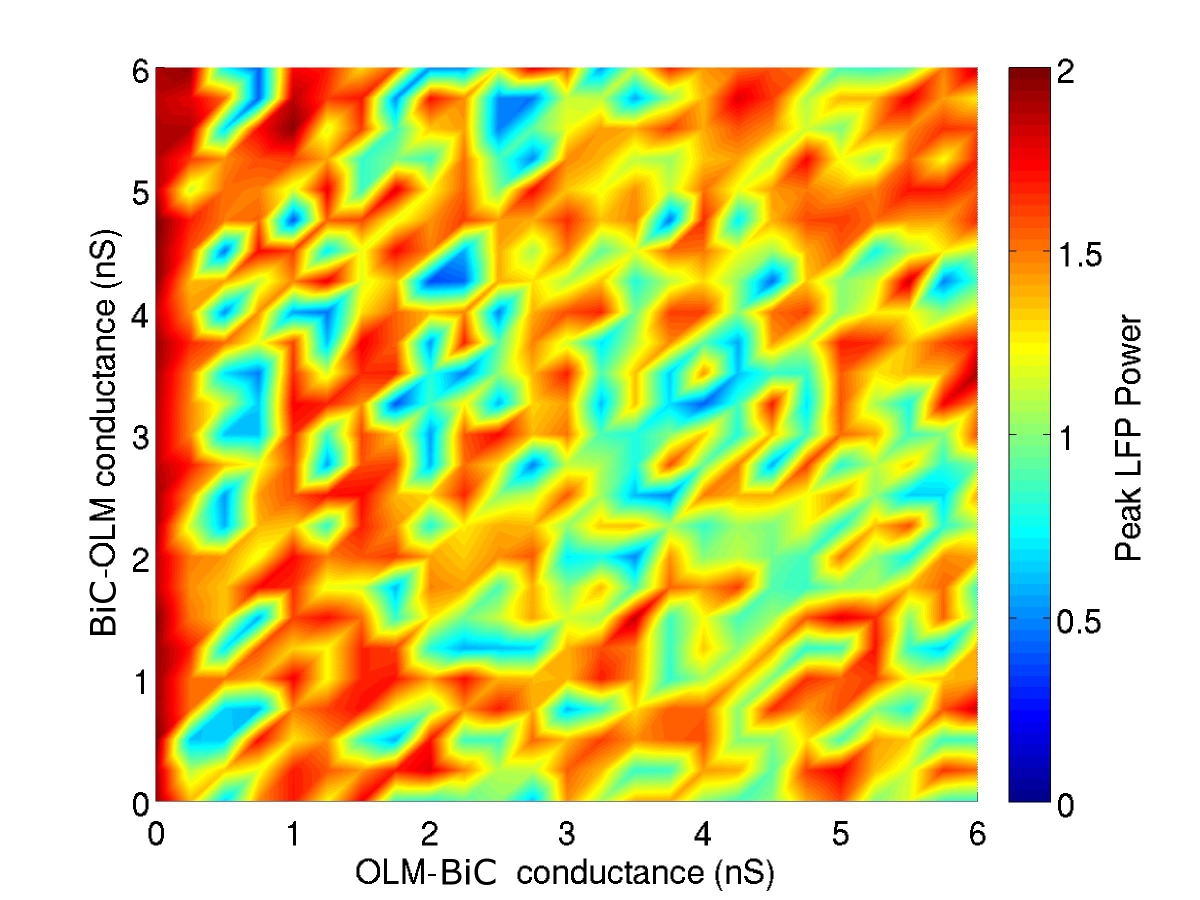

Supplement: Supplementary file 1 [file Presentation1.ZIP › FigS1d.jpg]

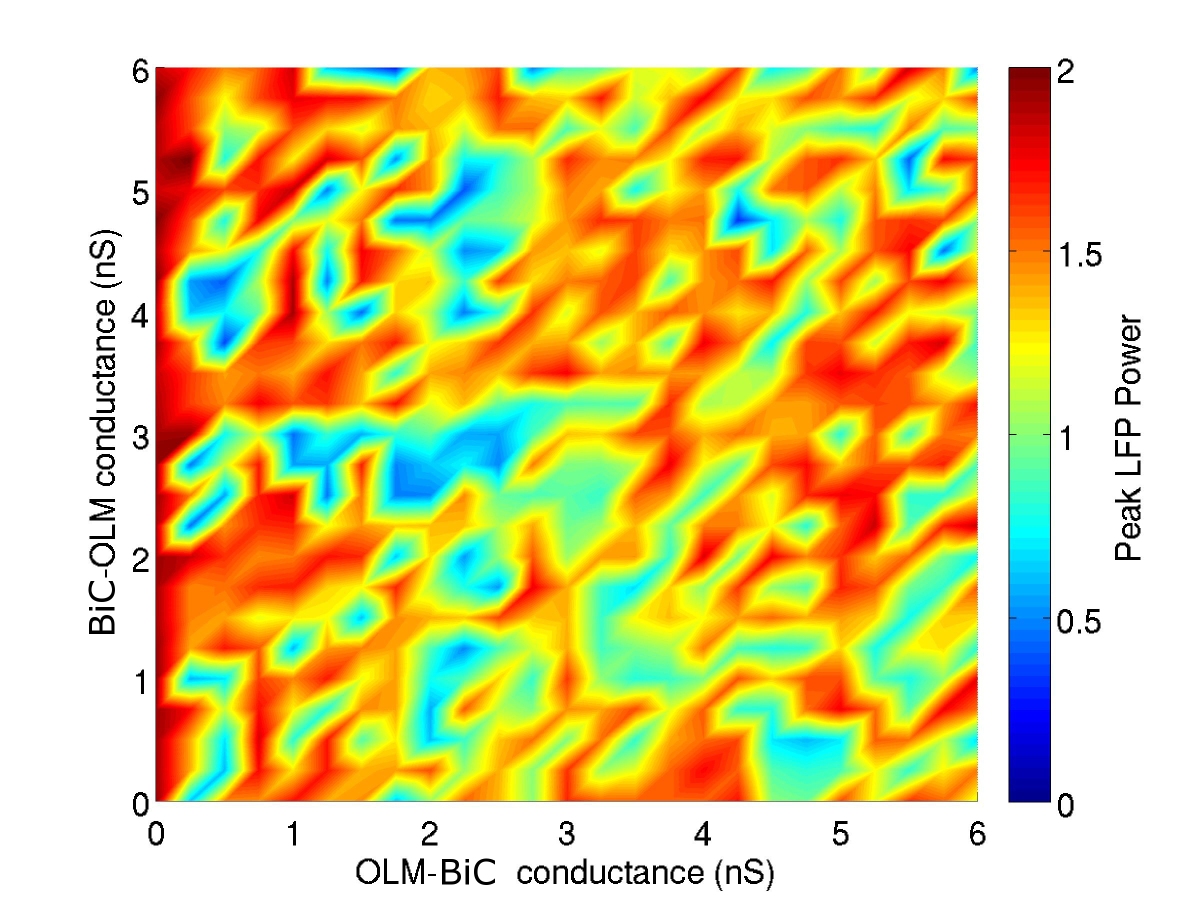

Supplement: Supplementary file 1 [file Presentation1.ZIP › FigS1e.jpg]

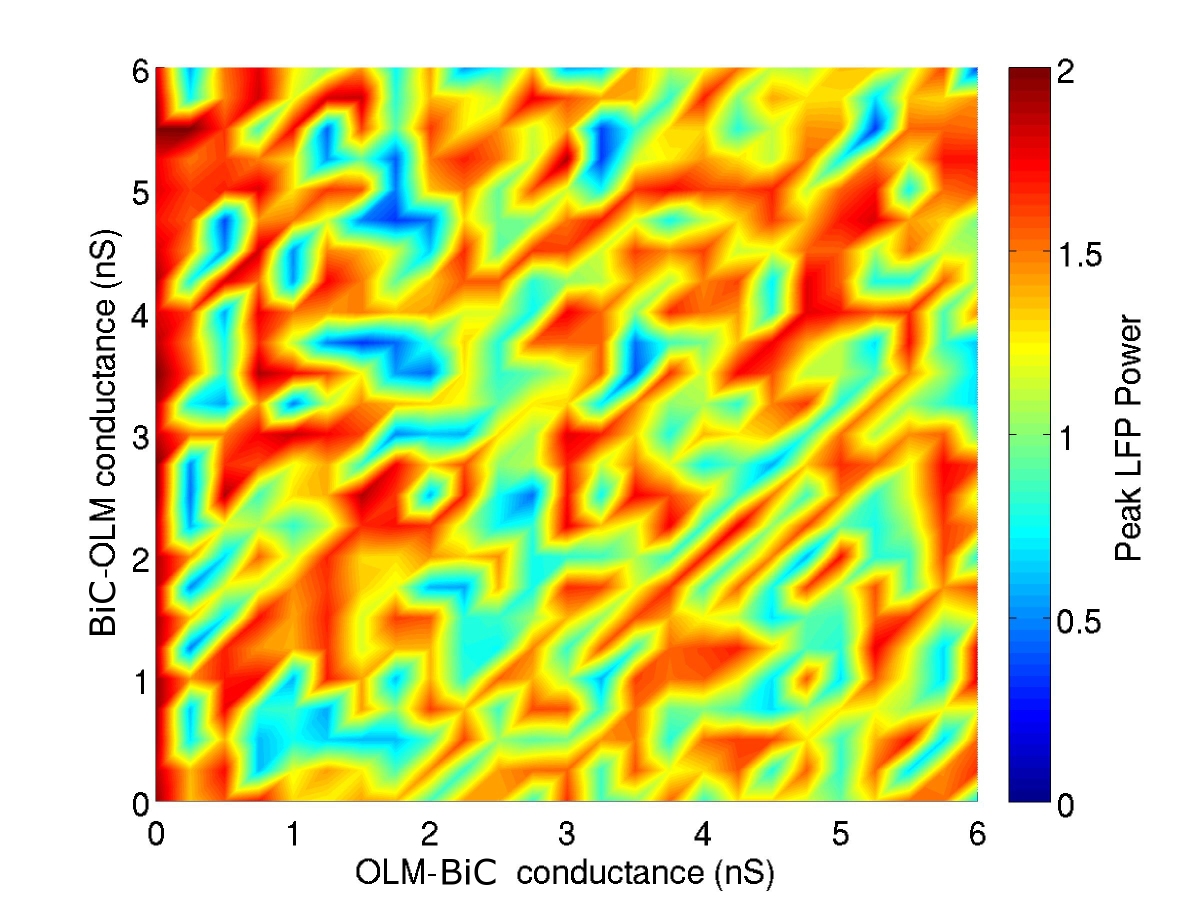

Supplement: Supplementary file 1 [file Presentation1.ZIP › FigS1f.jpg]

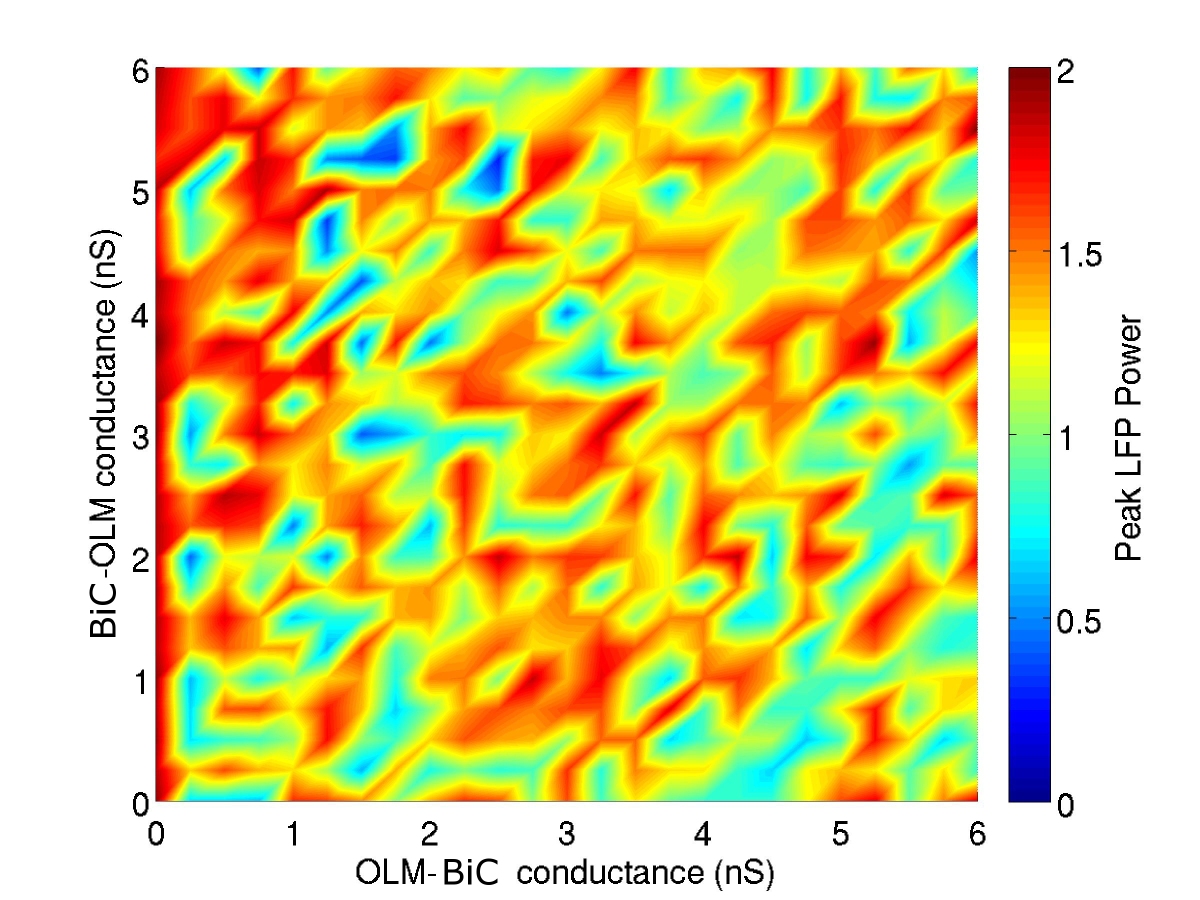

Supplement: Supplementary file 1 [file Presentation1.ZIP › FigS1g.jpg]

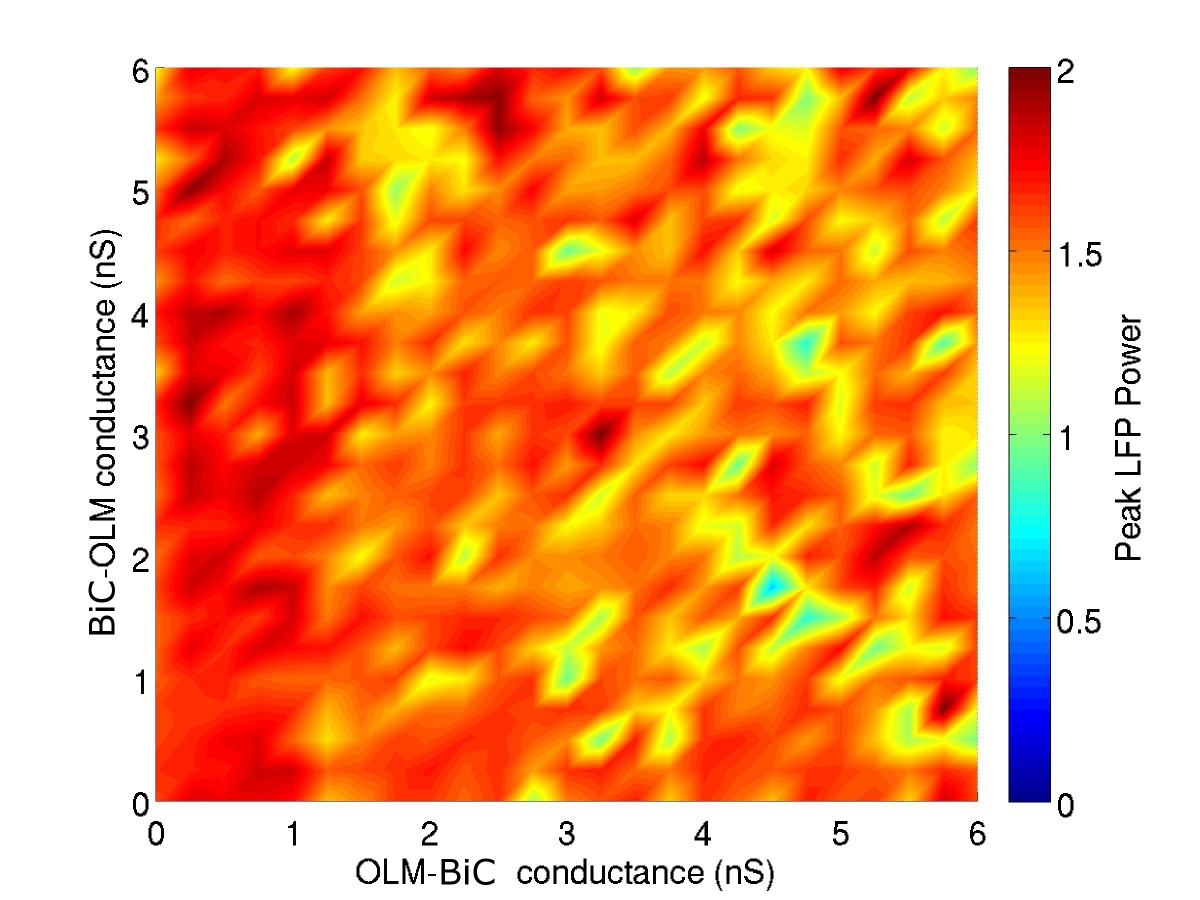

Supplement: Supplementary file 1 [file Presentation1.ZIP › FigS1h.jpg]

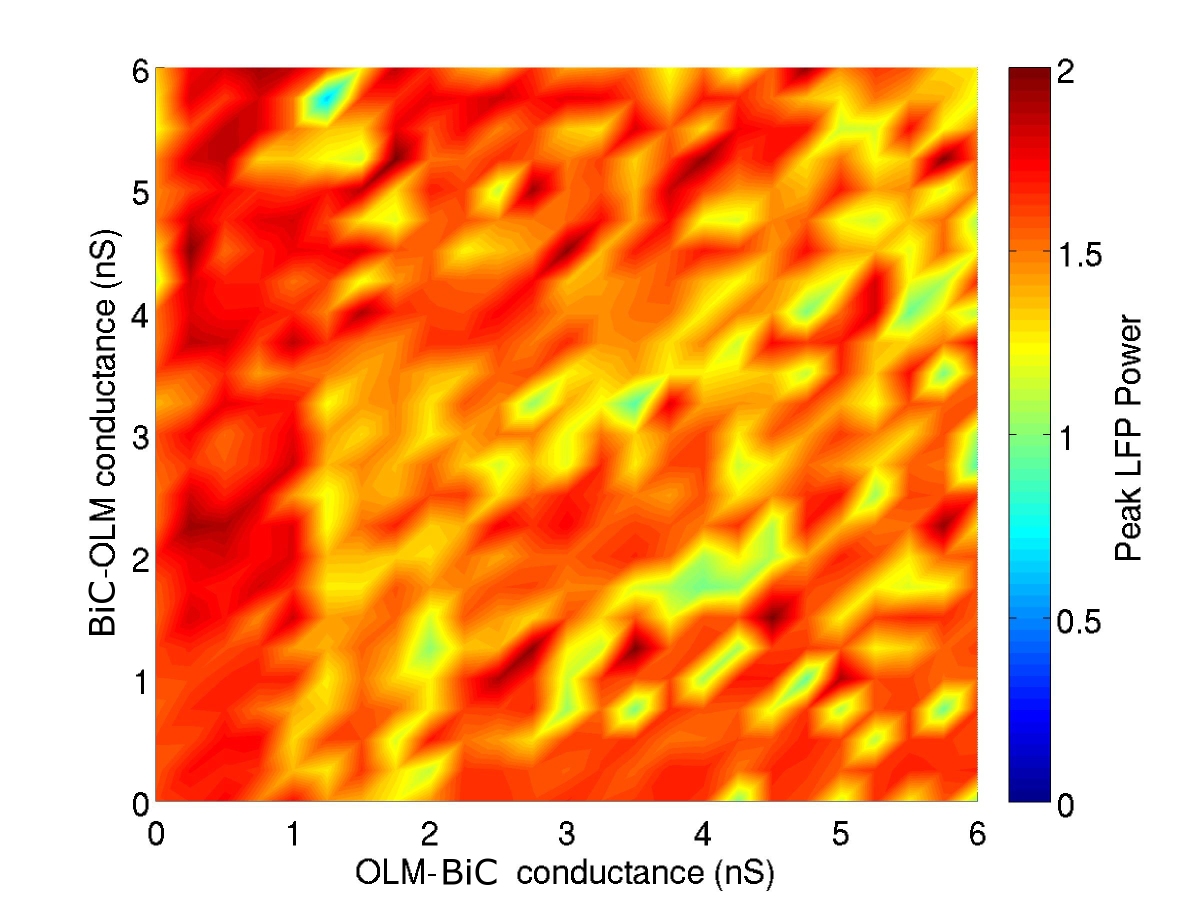

Supplement: Supplementary file 1 [file Presentation1.ZIP › FigS1i.jpg]

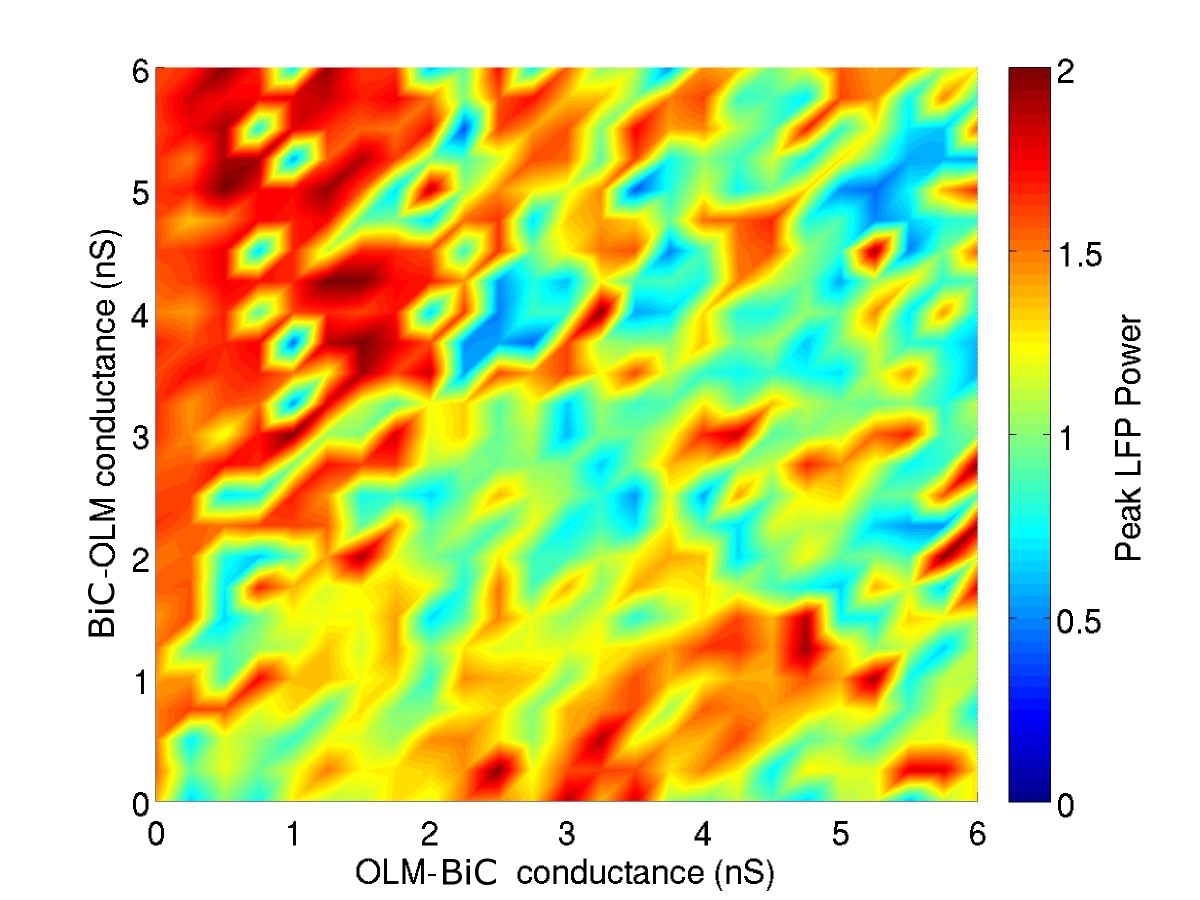

Supplement: Supplementary file 1 [file Presentation1.ZIP › FigS1j.jpg]

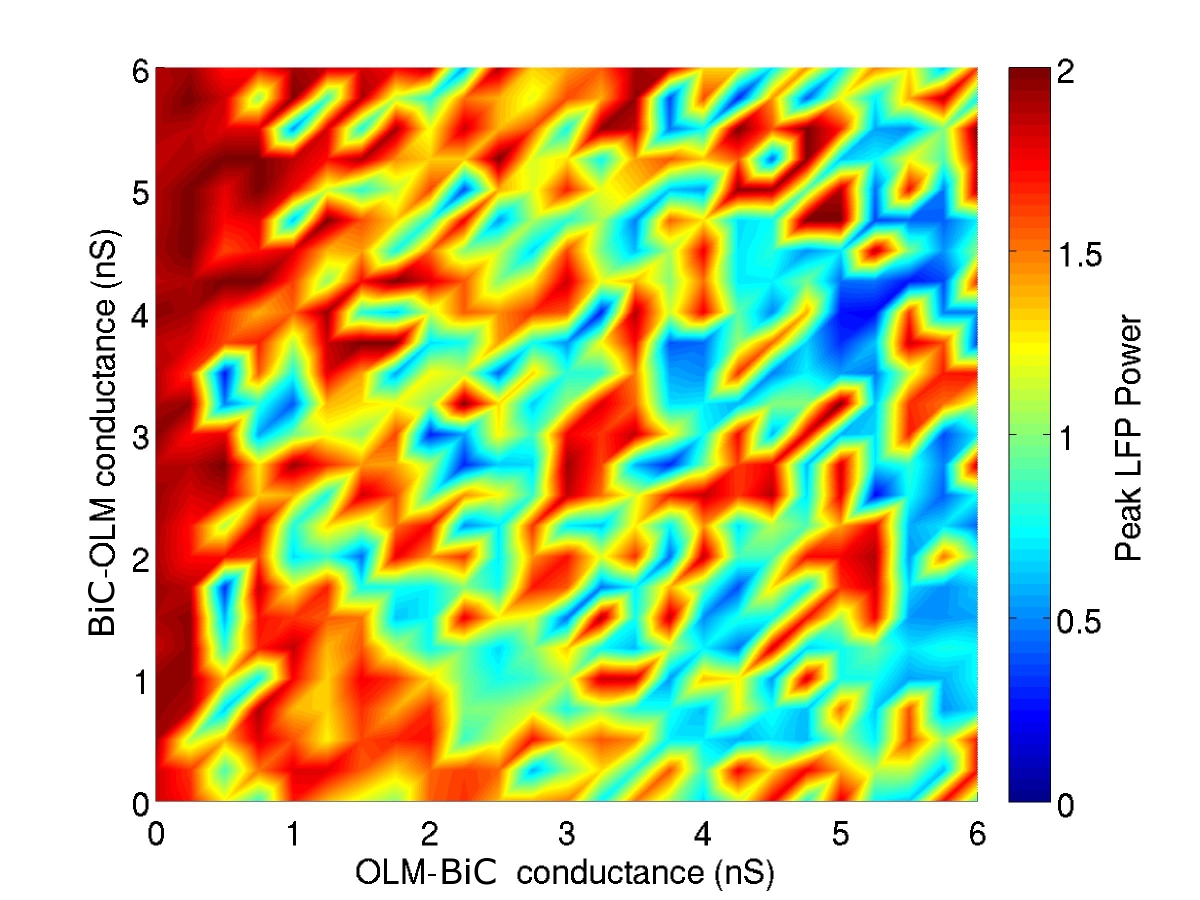

Supplement: Supplementary file 1 [file Presentation1.ZIP › FigS1k.jpg]

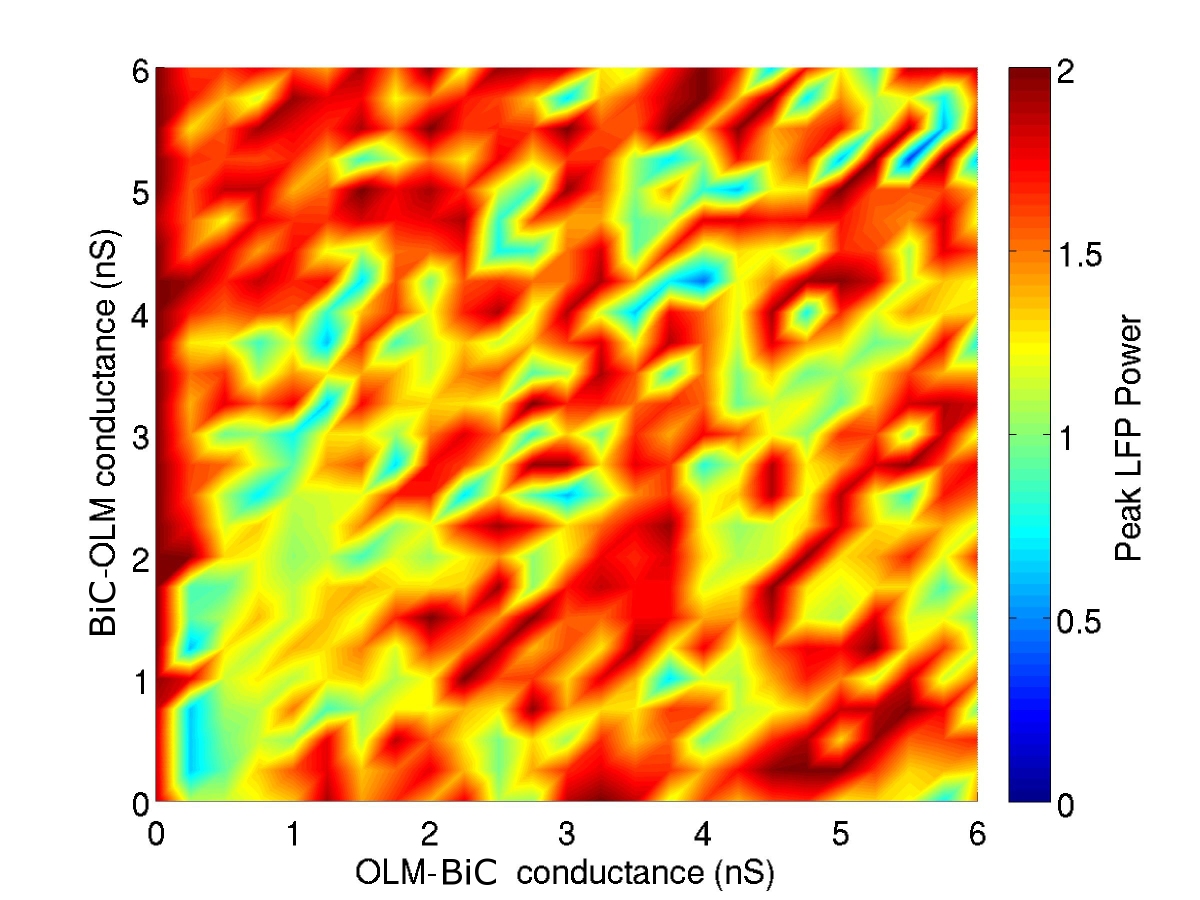

Supplement: Supplementary file 1 [file Presentation1.ZIP › FigS1l.jpg]

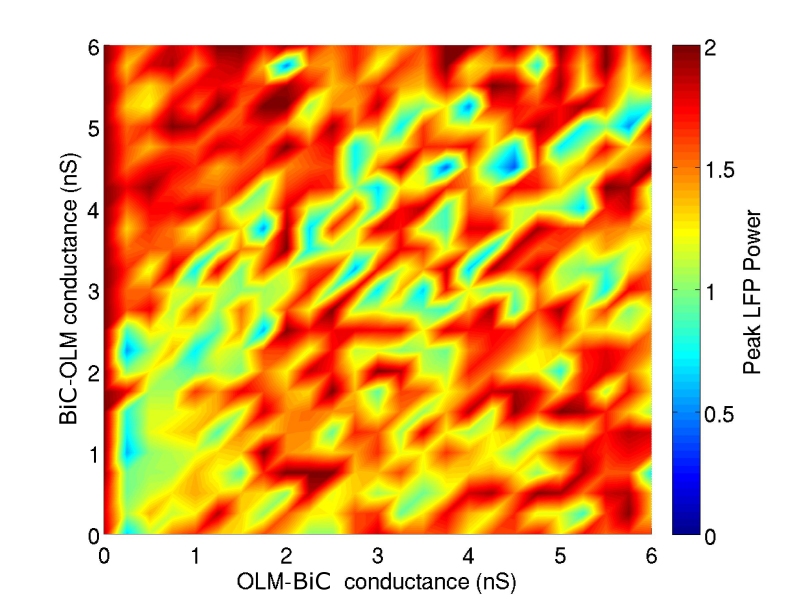

Supplement: Supplementary file 1 [file Presentation1.ZIP › FigS1m.jpg]

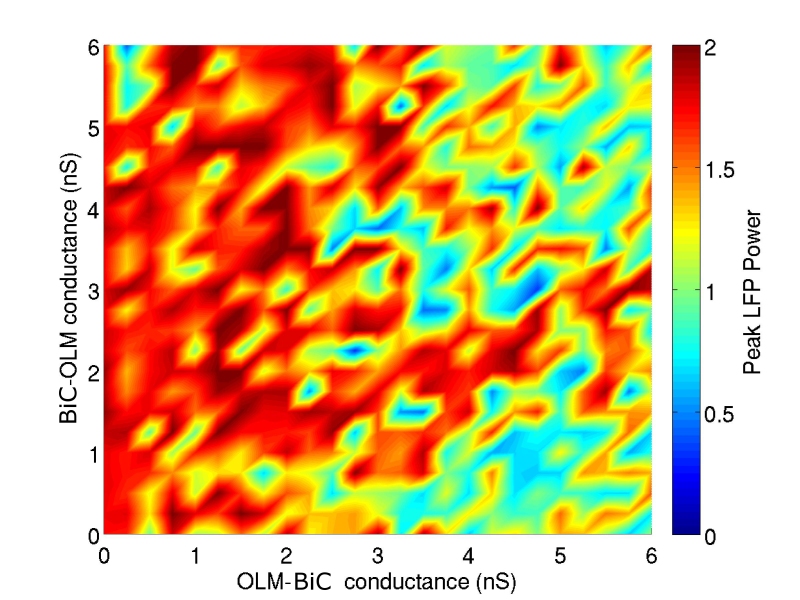

Supplement: Supplementary file 1 [file Presentation1.ZIP › FigS1n.jpg]

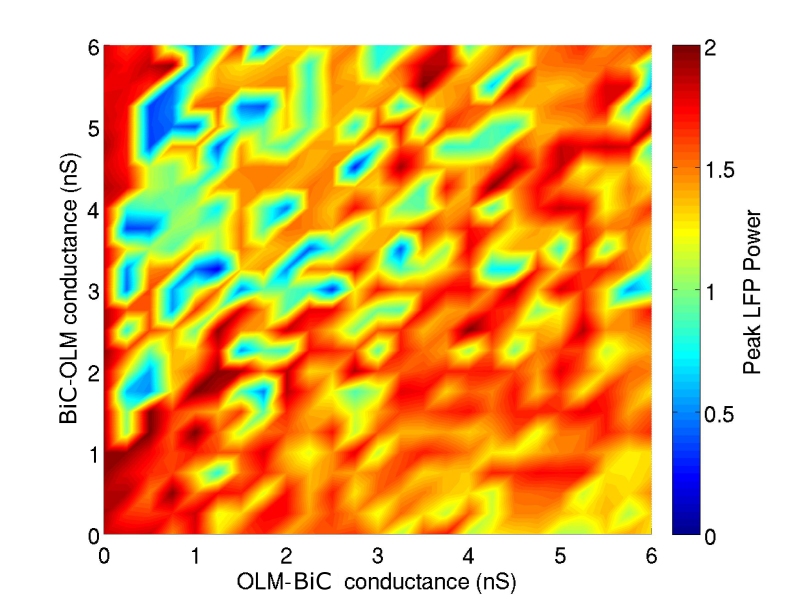

Supplement: Supplementary file 1 [file Presentation1.ZIP › FigS1o.jpg]

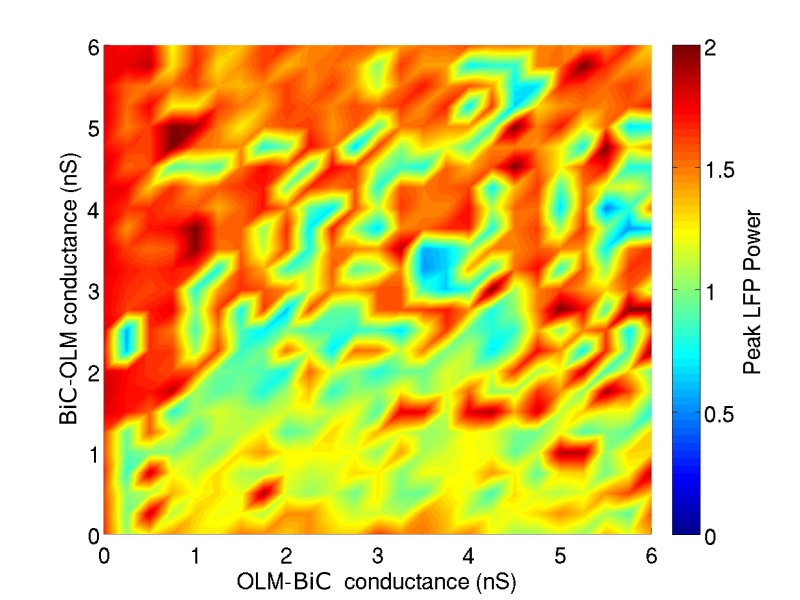

Supplement: Supplementary file 1 [file Presentation1.ZIP › FigS1p.jpg]

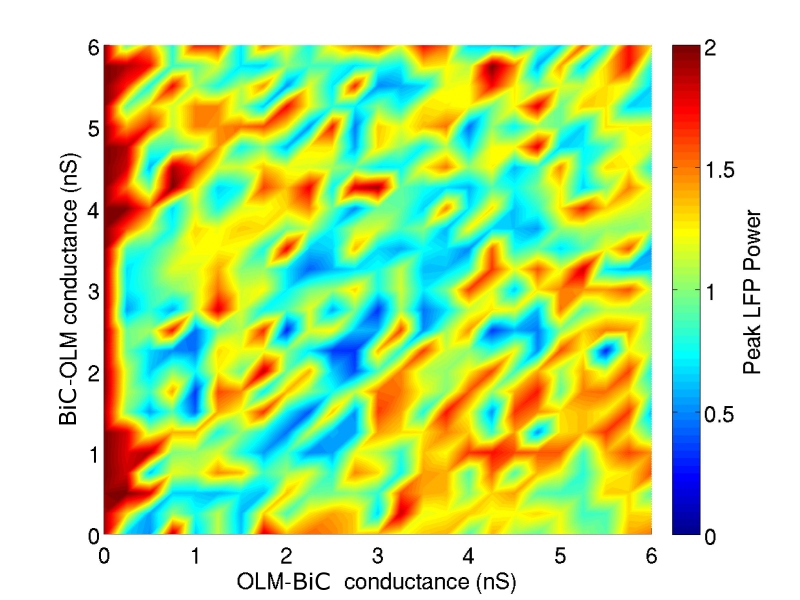

Supplement: Supplementary file 1 [file Presentation1.ZIP › FigS1q.jpg]

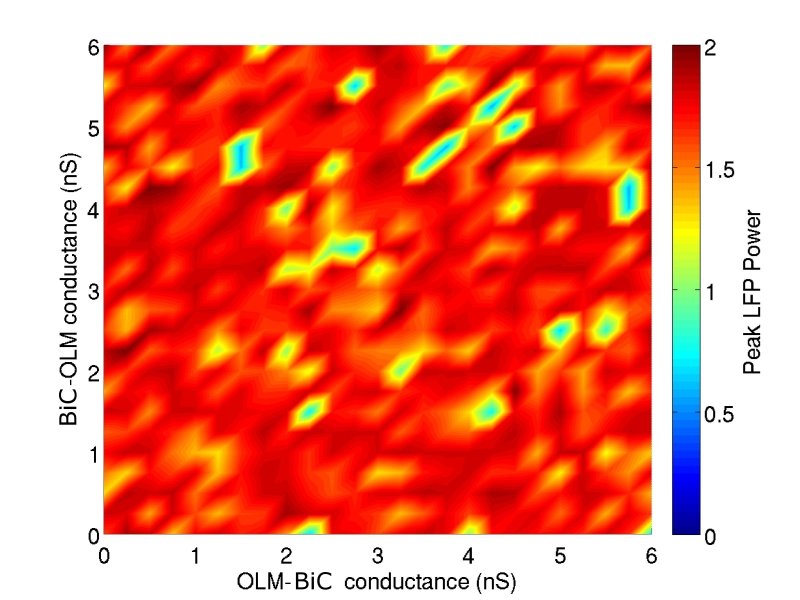

Supplement: Supplementary file 1 [file Presentation1.ZIP › FigS2a.jpg]

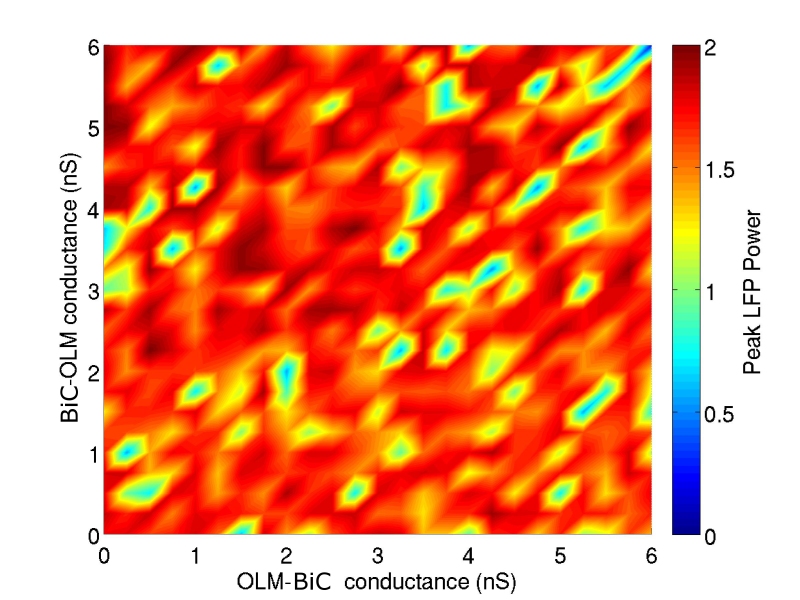

Supplement: Supplementary file 1 [file Presentation1.ZIP › FigS2b.jpg]

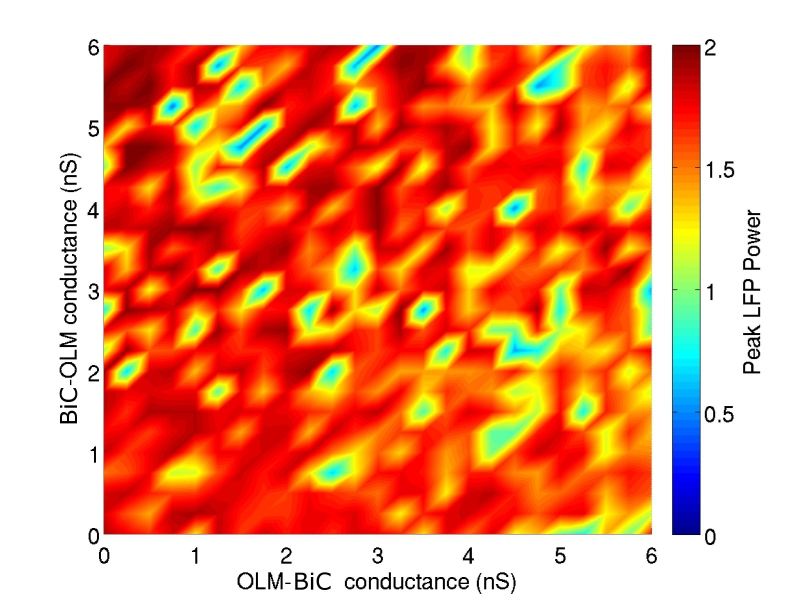

Supplement: Supplementary file 1 [file Presentation1.ZIP › FigS2c.jpg]

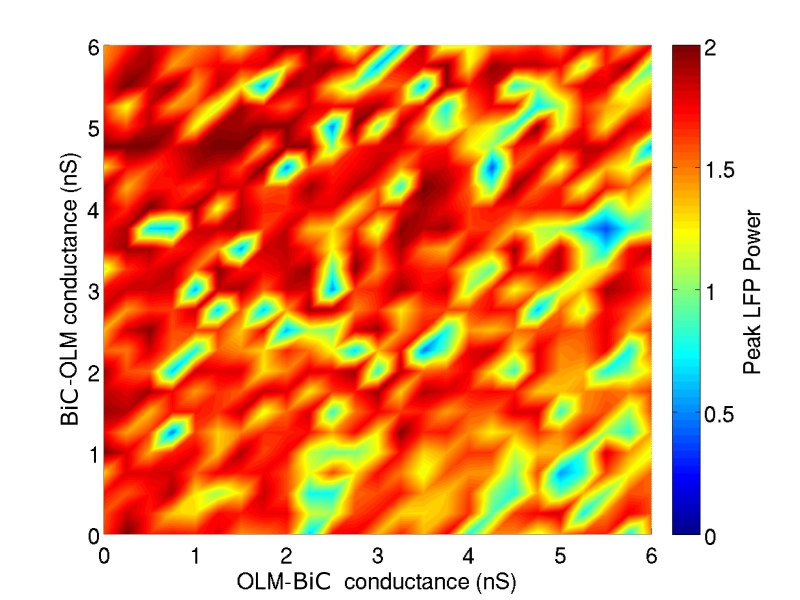

Supplement: Supplementary file 1 [file Presentation1.ZIP › FigS2d.jpg]

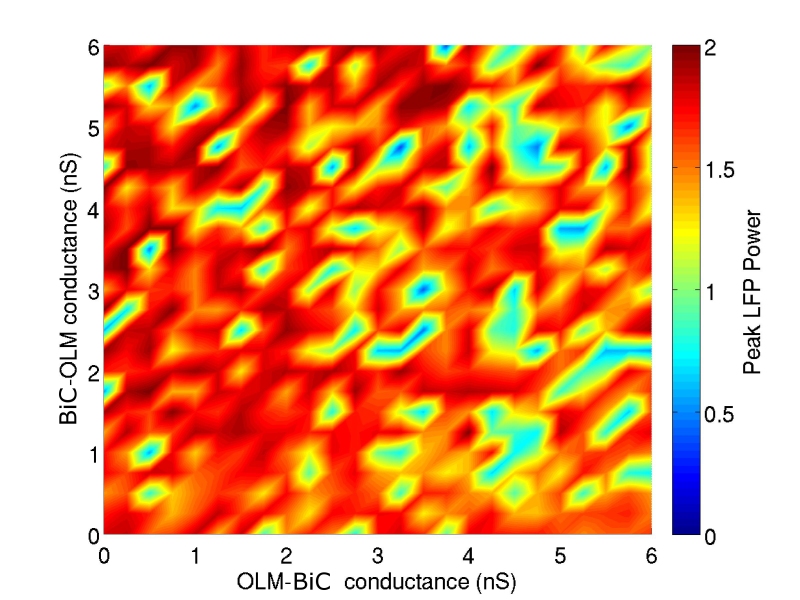

Supplement: Supplementary file 1 [file Presentation1.ZIP › FigS2e.jpg]

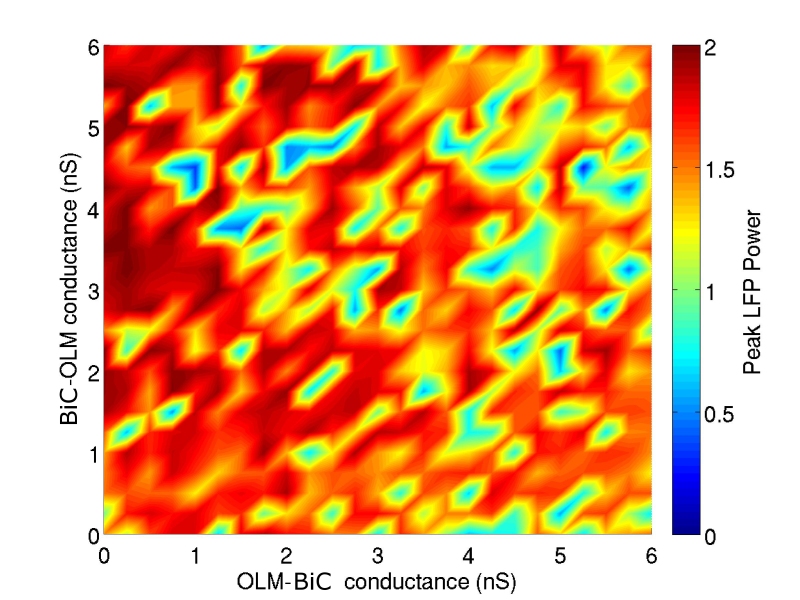

Supplement: Supplementary file 1 [file Presentation1.ZIP › FigS2f.jpg]

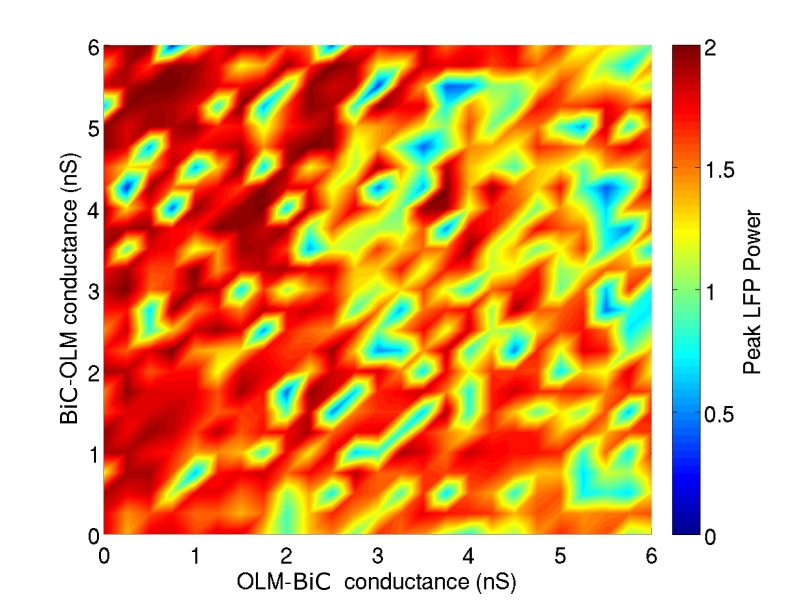

Supplement: Supplementary file 1 [file Presentation1.ZIP › FigS2g.jpg]

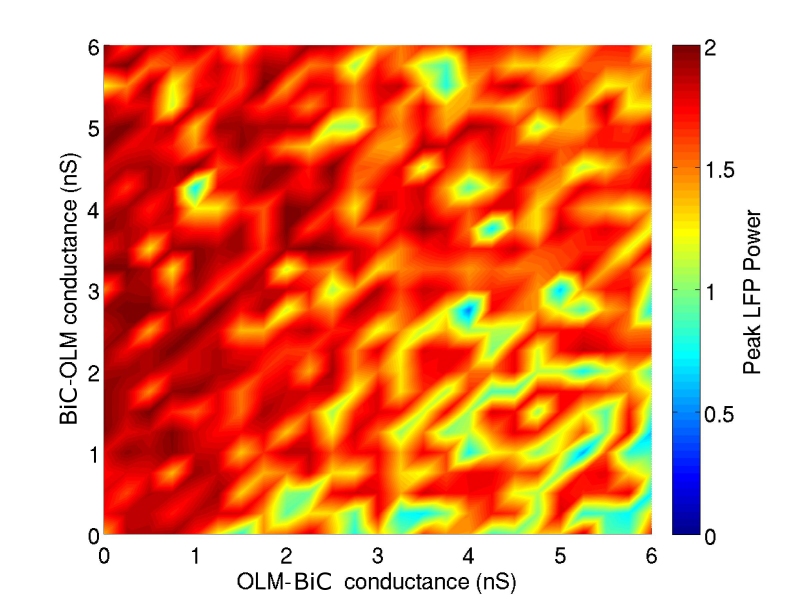

Supplement: Supplementary file 1 [file Presentation1.ZIP › FigS2h.jpg]

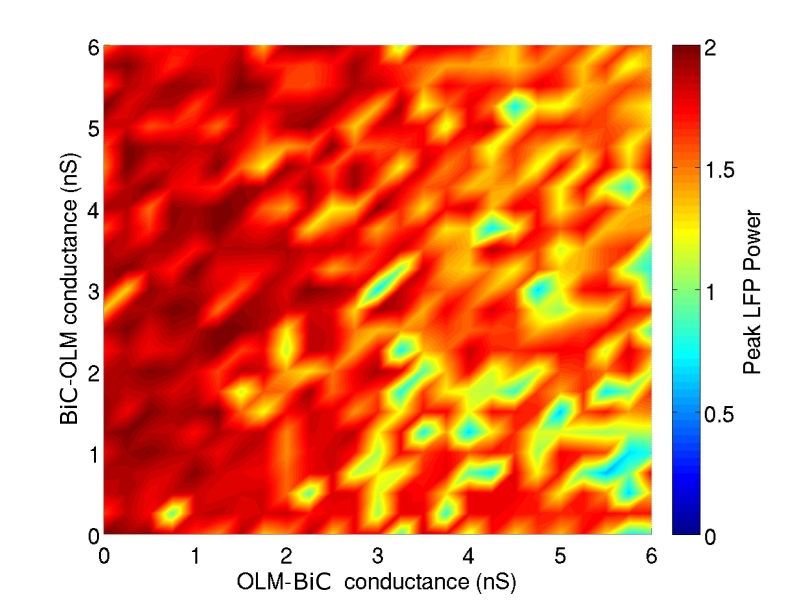

Supplement: Supplementary file 1 [file Presentation1.ZIP › FigS2i.jpg]

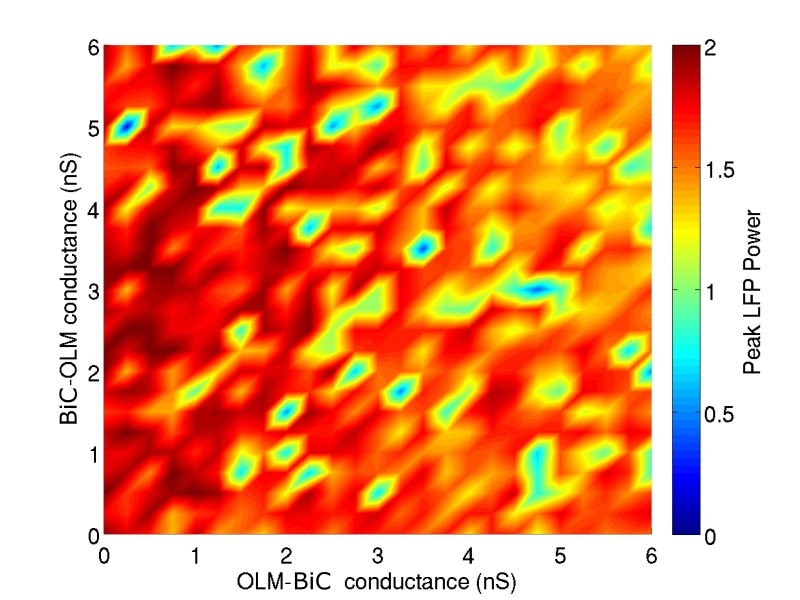

Supplement: Supplementary file 1 [file Presentation1.ZIP › FigS2j.jpg]

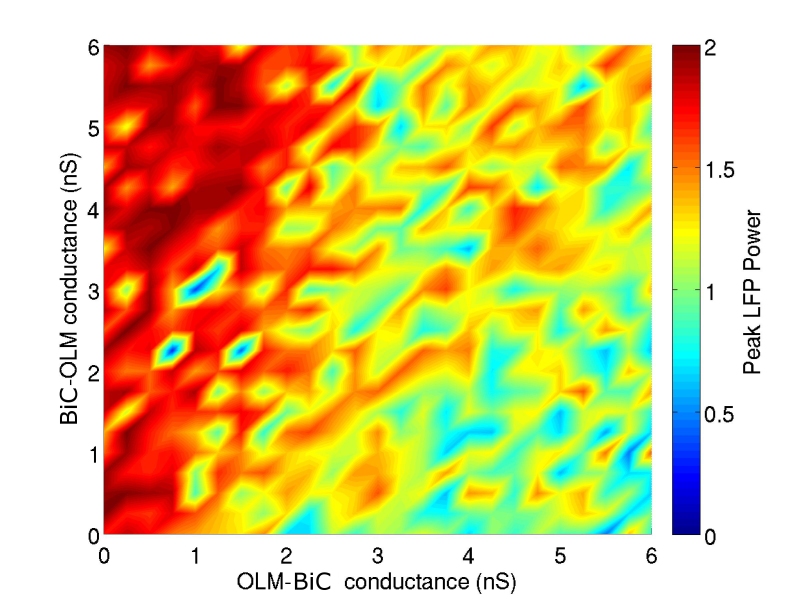

Supplement: Supplementary file 1 [file Presentation1.ZIP › FigS2k.jpg]

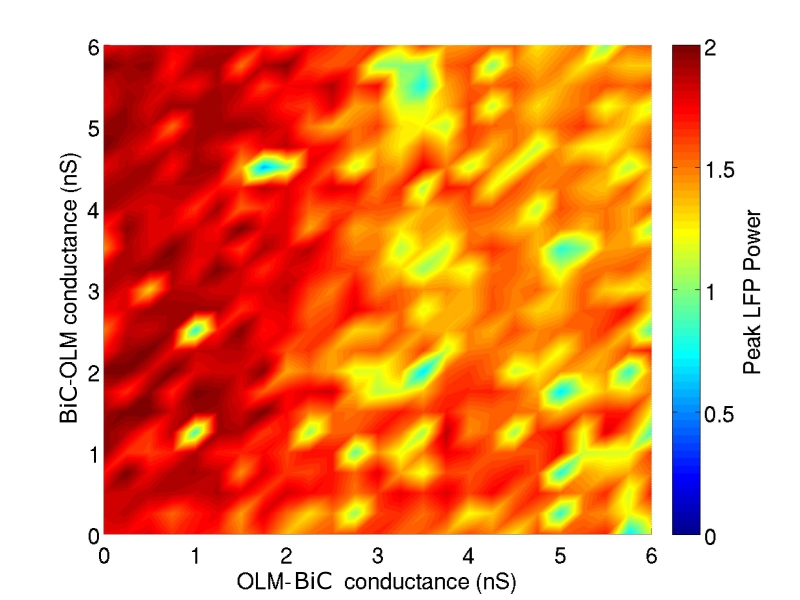

Supplement: Supplementary file 1 [file Presentation1.ZIP › FigS2l.jpg]

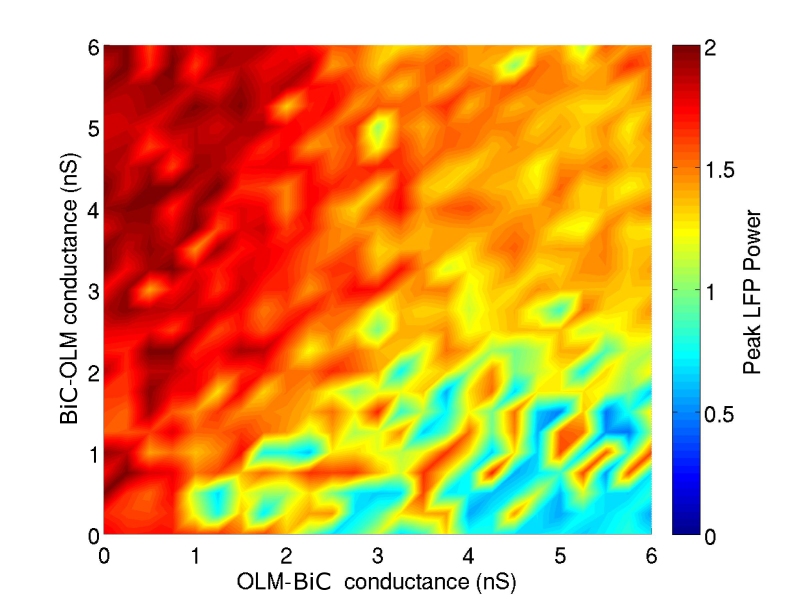

Supplement: Supplementary file 1 [file Presentation1.ZIP › FigS2m.jpg]

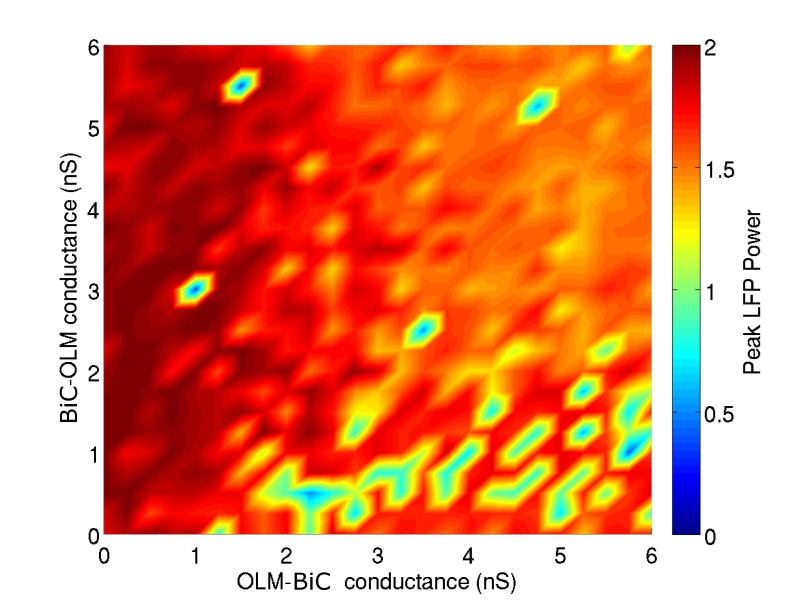

Supplement: Supplementary file 1 [file Presentation1.ZIP › FigS2n.jpg]

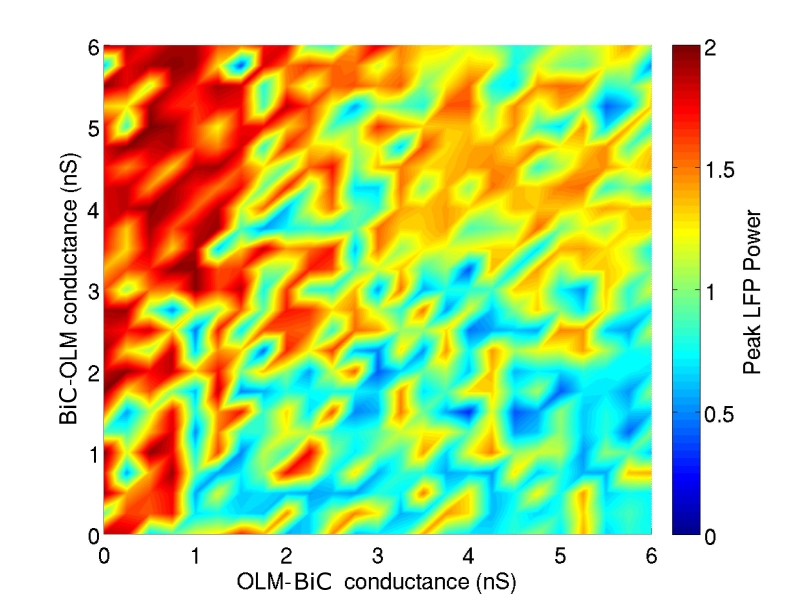

Supplement: Supplementary file 1 [file Presentation1.ZIP › FigS2o.jpg]

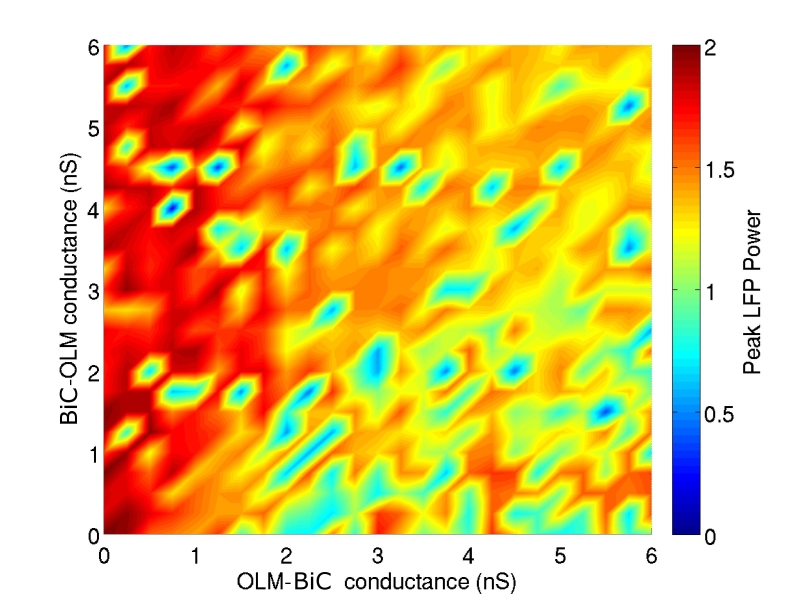

Supplement: Supplementary file 1 [file Presentation1.ZIP › FigS2p.jpg]

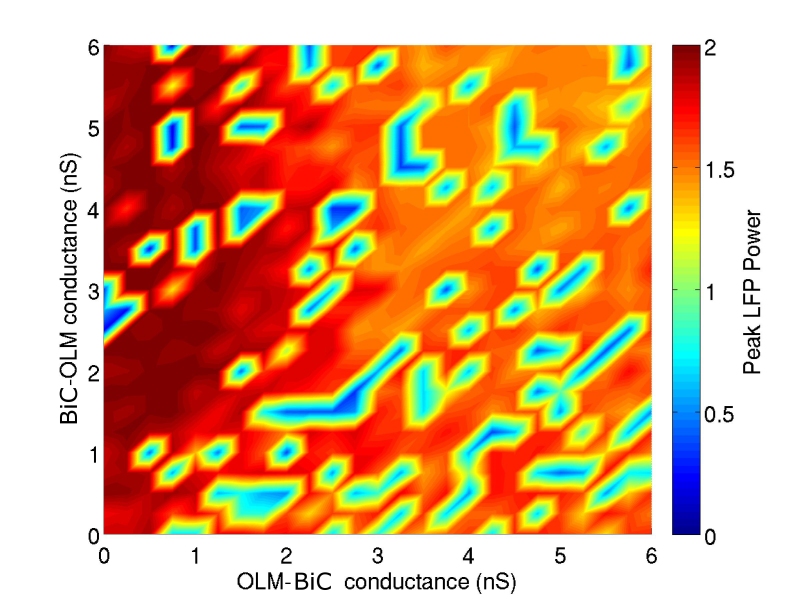

Supplement: Supplementary file 1 [file Presentation1.ZIP › FigS2q.jpg]

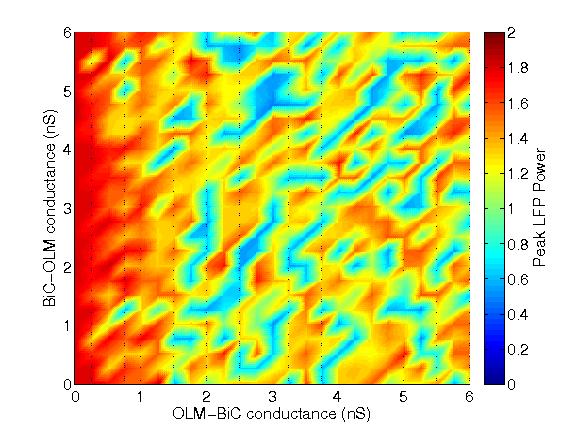

Supplement: Supplementary file 1 [file Presentation1.ZIP › FigS3a.jpg]

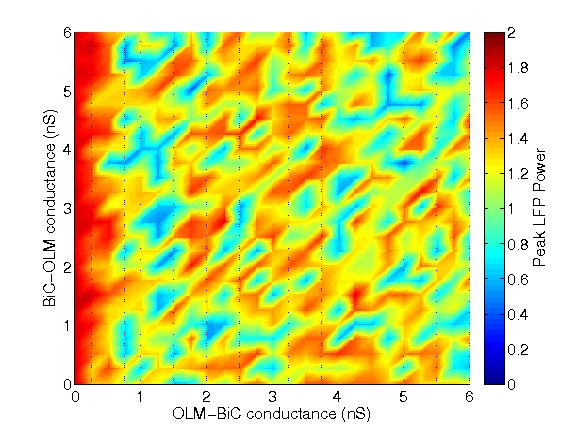

Supplement: Supplementary file 1 [file Presentation1.ZIP › FigS3b.jpg]

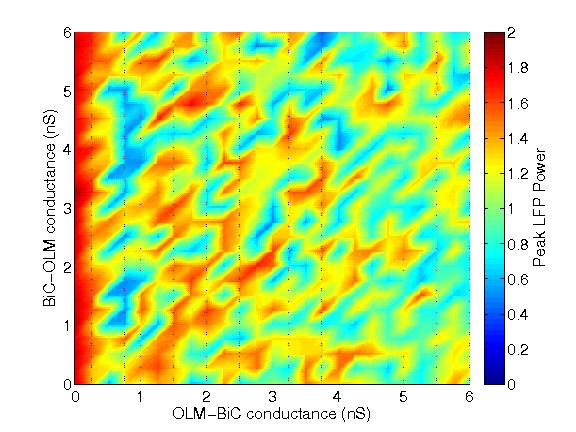

Supplement: Supplementary file 1 [file Presentation1.ZIP › FigS3c.jpg]

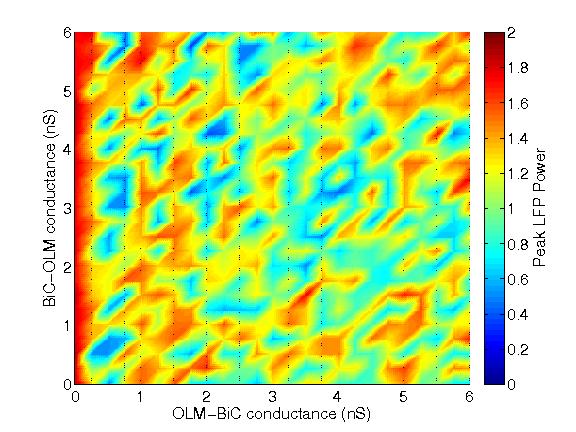

Supplement: Supplementary file 1 [file Presentation1.ZIP › FigS3d.jpg]

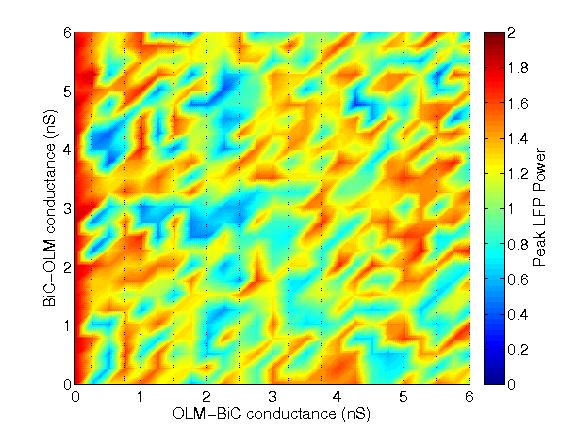

Supplement: Supplementary file 1 [file Presentation1.ZIP › FigS3e.jpg]

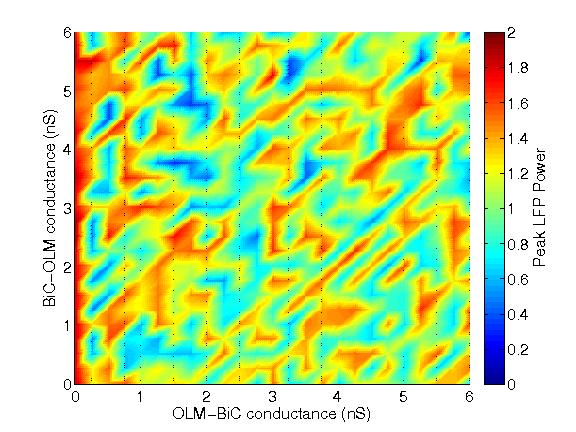

Supplement: Supplementary file 1 [file Presentation1.ZIP › FigS3f.jpg]

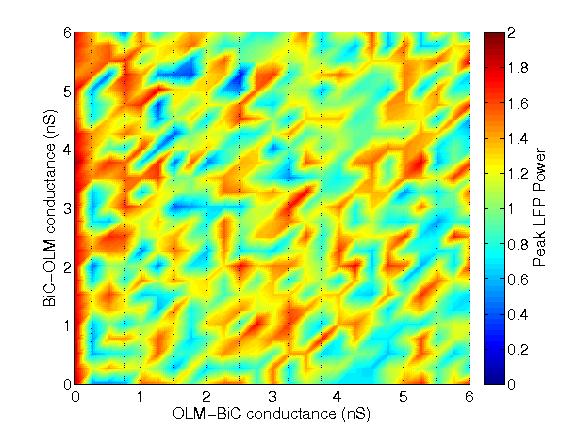

Supplement: Supplementary file 1 [file Presentation1.ZIP › FigS3g.jpg]

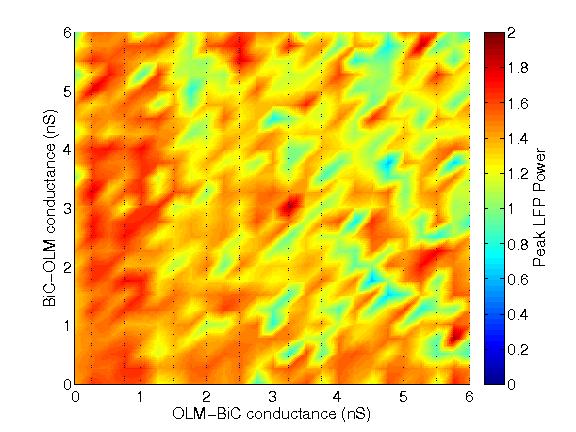

Supplement: Supplementary file 1 [file Presentation1.ZIP › FigS3h.jpg]

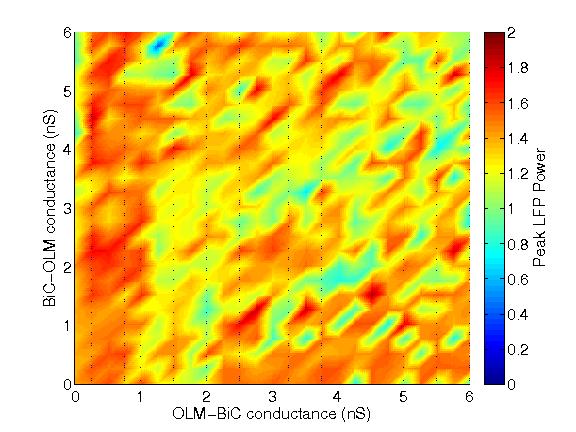

Supplement: Supplementary file 1 [file Presentation1.ZIP › FigS3i.jpg]

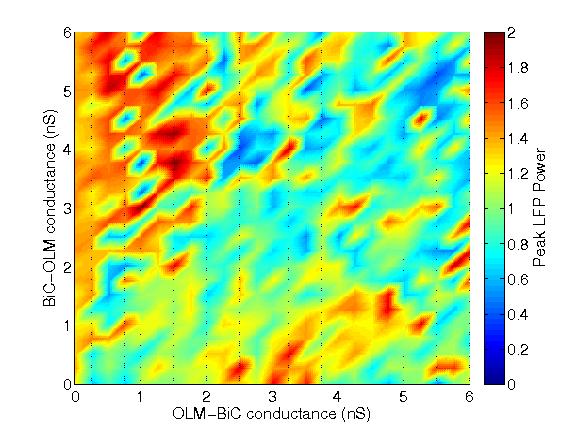

Supplement: Supplementary file 1 [file Presentation1.ZIP › FigS3j.jpg]

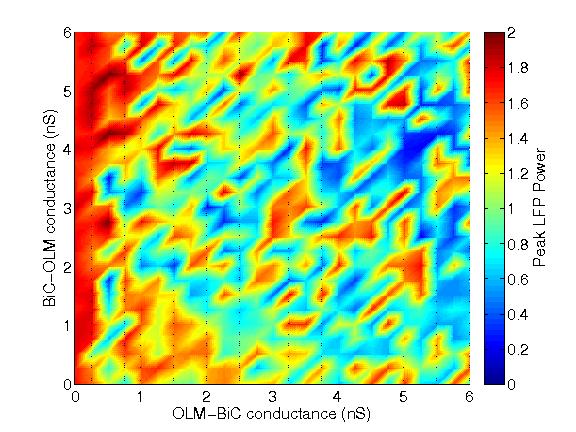

Supplement: Supplementary file 1 [file Presentation1.ZIP › FigS3k.jpg]

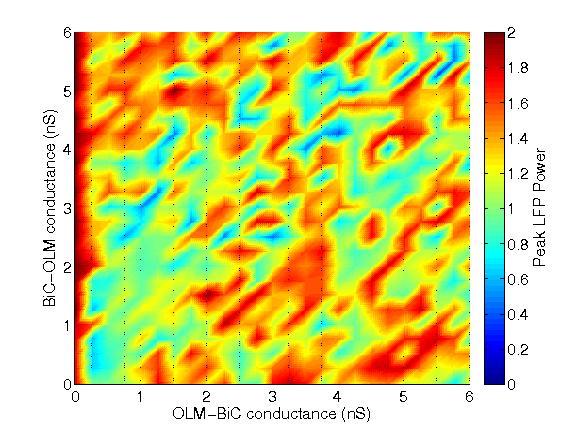

Supplement: Supplementary file 1 [file Presentation1.ZIP › FigS3l.jpg]

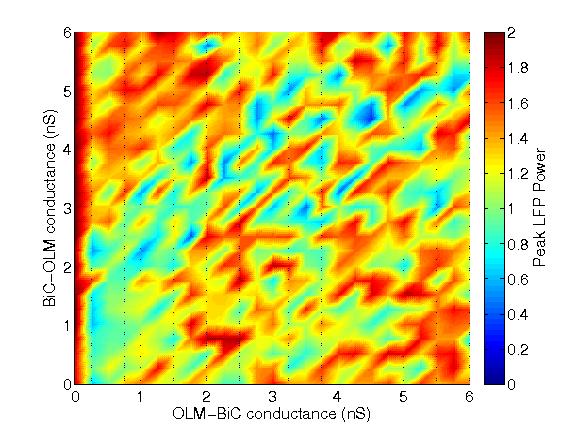

Supplement: Supplementary file 1 [file Presentation1.ZIP › FigS3m.jpg]

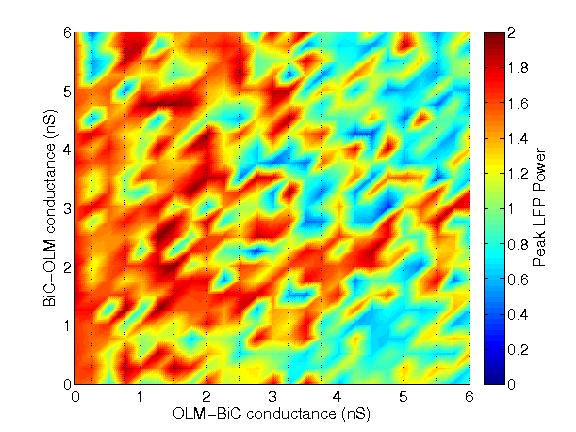

Supplement: Supplementary file 1 [file Presentation1.ZIP › FigS3n.jpg]

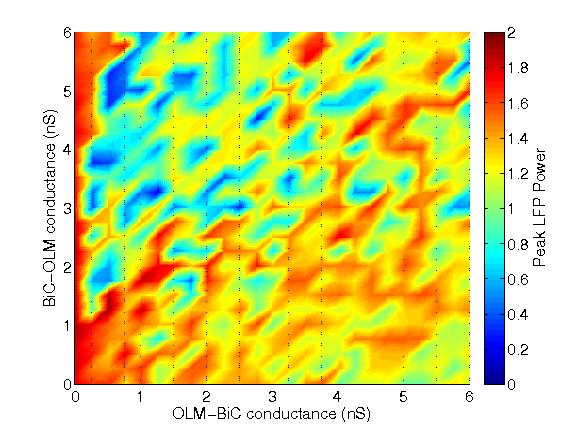

Supplement: Supplementary file 1 [file Presentation1.ZIP › FigS3o.jpg]

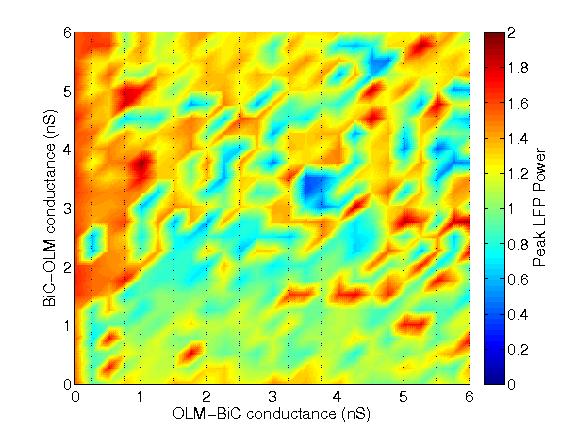

Supplement: Supplementary file 1 [file Presentation1.ZIP › FigS3p.jpg]

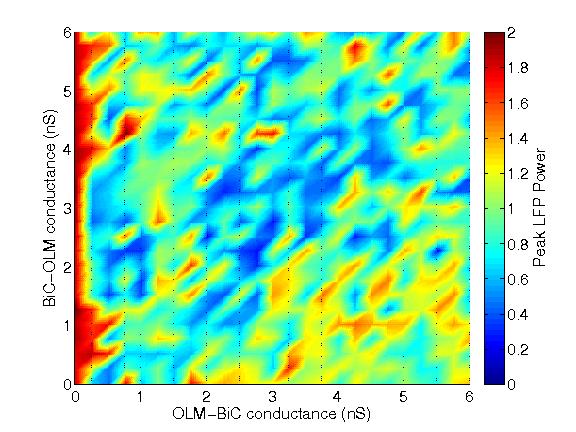

Supplement: Supplementary file 1 [file Presentation1.ZIP › FigS3q.jpg]
