# Supplementary material for: Network models provide insights into how oriens–lacunosum-moleculare and bistratified cell interactions influence the power of local hippocampal CA1 theta oscillations
Source: Front Syst Neurosci. 2015 Aug 7;9:110. doi: 10.3389/fnsys.2015.00110 (PMC4528165; doi:10.3389/fnsys.2015.00110)
Supplement: Supplementary file 2 [file DataSheet1.PDF]

Supplementary Material: Network models provide insight into how oriens–lacunosum-moleculare (OLM) and bistratified cell (BSC) interactions influence the power of local hippocampal CA1 theta oscillations

Katie A. Ferguson [1,2,*], Carey Y.L. Huh [3], Bénédicte Amilhon [3], Frédéric Manseau [3], Sylvain Williams [3] and Frances K. Skinner [1,4]

[1] *Division of Fundamental Neurobiology, Toronto Western Research Institute, University Health Network, Toronto, ON, Canada*
[2] *Department of Physiology, University of Toronto, Toronto, ON, Canada*
[3] *Department of Psychiatry, Douglas Mental Health University Institute, McGill University, Montreal, QC, Canada*
[4] *Departments of Medicine (Neurology) and Physiology, University of Toronto, Toronto, ON, Canada*

Correspondence*:
Katie A. Ferguson
Division of Fundamental Neurobiology, Toronto Western Research Institute, University Health Network
Krembil Discovery Tower, Toronto Western Hospital, 60 Leonard Avenue, 7KD410, Toronto, ON, Canada, M5T 2S8, katie.ferguson@utoronto.ca

1 SUPPLEMENTARY INFORMATION

1.1 PREVIOUSLY PUBLISHED PV+ FAST-SPIKING MODEL

We created a CA1 fast-spiking parvalbumin-positive (PV+) interneuron model in **Ferguson et al.** (2013). As with the somatostatin-positive (SOM+) cell model (described in the main text), we used experimental recordings to determine the intrinsic spike shape and firing properties of the cells, and based the individual model on the modified **Izhikevich** (2003) model, as described in Equation 1 (of the main text). In this model, our constraints were based on properties determined from CA1 fast-spiking PV+ interneurons, recorded in the stratum oriens layer. For convenience, the model parameters (from **Ferguson et al.**, 2013) are given in Table S1.

2 SUPPLEMENTARY TABLES

Table S1. PV+ model parameters

Parameter	PV+ model
C_m (pF)	90
v_r (mV)	-60.6
v_t (mV)	-43.1
v_{peak} (mV)	-2.5
a (ms^{-1})	0.1
b (nS)	-0.1
c (mV)	-67
d (pA)	0.1
k_{low} (nS/mV)	1.7
k_{high} (nS/mV)	14
I_{shift} (pA)	0

3 SUPPLEMENTARY FIGURES

(a) $c_{OLM,BiC} = 0.01$ (b) $c_{OLM,BiC} = 0.03$ (c) $c_{OLM,BiC} = 0.05$ (d) $c_{OLM,BiC} = 0.07$

(e) $c_{OLM,BiC} = 0.09$ (f) $c_{OLM,BiC} = 0.11$ (g) $c_{OLM,BiC} = 0.13$ (h) $c_{OLM,BiC} = 0.15$

(i) $c_{OLM,BiC} = 0.17$ (j) $c_{OLM,BiC} = 0.19$ (k) $c_{OLM,BiC} = 0.21$ (l) $c_{OLM,BiC} = 0.23$

(m) $c_{OLM,BiC} = 0.25$ (n) $c_{OLM,BiC} = 0.27$ (o) $c_{OLM,BiC} = 0.29$ (p) $c_{OLM,BiC} = 0.31$

(q) $c_{OLM,BiC} = 0.33$

Figure S1: Peak local field potential (LFP) power of our network models for various connection probabilities.

(a) $c_{OLM,BiC} = 0.01$ (b) $c_{OLM,BiC} = 0.03$ (c) $c_{OLM,BiC} = 0.05$ (d) $c_{OLM,BiC} = 0.07$

(e) $c_{OLM,BiC} = 0.09$ (f) $c_{OLM,BiC} = 0.11$ (g) $c_{OLM,BiC} = 0.13$ (h) $c_{OLM,BiC} = 0.15$

(i) $c_{OLM,BiC} = 0.17$ (j) $c_{OLM,BiC} = 0.19$ (k) $c_{OLM,BiC} = 0.21$ (l) $c_{OLM,BiC} = 0.23$

(m) $c_{OLM,BiC} = 0.25$ (n) $c_{OLM,BiC} = 0.27$ (o) $c_{OLM,BiC} = 0.29$ (p) $c_{OLM,BiC} = 0.31$

(q) $c_{OLM,BiC} = 0.33$

Figure S2: Peak LFP power of our network models with basket and axo-axonic cell (BC/AAC) silenced for various connection probabilities.

(a) $c_{OLM,BiC} = 0.01$ (b) $c_{OLM,BiC} = 0.03$ (c) $c_{OLM,BiC} = 0.05$ (d) $c_{OLM,BiC} = 0.07$

(e) $c_{OLM,BiC} = 0.09$ (f) $c_{OLM,BiC} = 0.11$ (g) $c_{OLM,BiC} = 0.13$ (h) $c_{OLM,BiC} = 0.15$

(i) $c_{OLM,BiC} = 0.17$ (j) $c_{OLM,BiC} = 0.19$ (k) $c_{OLM,BiC} = 0.21$ (l) $c_{OLM,BiC} = 0.23$

(m) $c_{OLM,BiC} = 0.25$ (n) $c_{OLM,BiC} = 0.27$ (o) $c_{OLM,BiC} = 0.29$ (p) $c_{OLM,BiC} = 0.31$

(q) $c_{OLM,BiC} = 0.33$

Figure S3: Peak LFP power of our network models for increasing LFP weights with distance from the soma, for various connection probabilities.

REFERENCES

Ferguson, K. A., Huh, C. Y. L., Amilhon, B., Williams, S., and Skinner, F. K. (2013), Experimentally constrained CA1 fast-firing parvalbumin-positive interneuron network models exhibit sharp transitions into coherent high frequency rhythms, *Frontiers in Computational Neuroscience*, 7, 144, doi:10.3389/fncom.2013.00144

Izhikevich, E. M. (2003), Simple model of spiking neurons, *IEEE Transactions on Neural Networks / a Publication of the IEEE Neural Networks Council*, 14, 6, 1569–1572, doi:10.1109/TNN.2003.820440
